# eIF4EBP3L Acts as a Gatekeeper of TORC1 In Activity-Dependent Muscle Growth by Specifically Regulating Mef2ca Translational Initiation

**DOI:** 10.1371/journal.pbio.1001679

**Published:** 2013-10-15

**Authors:** Orli Yogev, Victoria C. Williams, Yaniv Hinits, Simon M. Hughes

**Affiliations:** Randall Division of Cell and Molecular Biophysics, King's College London, London, United Kingdom; The Wellcome Trust Sanger Institute, United Kingdom

## Abstract

Muscle fiber size is activity-dependent and clinically important in ageing, bed-rest, and cachexia, where muscle weakening leads to disability, prolonged recovery times, and increased costs. Inactivity causes muscle wasting by triggering protein degradation and may simultaneously prevent protein synthesis. During development, muscle tissue grows by several mechanisms, including hypertrophy of existing fibers. As in other tissues, the TOR pathway plays a key role in promoting muscle protein synthesis by inhibition of eIF4EBPs (eukaryotic Initiation Factor 4E Binding Proteins), regulators of the translational initiation. Here, we tested the role of TOR-eIF4EBP in a novel zebrafish muscle inactivity model. Inactivity triggered up-regulation of eIF4EBP3L (a zebrafish homolog of eIF4EBP3) and diminished myosin and actin content, myofibrilogenesis, and fiber growth. The changes were accompanied by preferential reduction of the muscle transcription factor Mef2c, relative to Myod and Vinculin. Polysomal fractionation showed that Mef2c decrease was due to reduced translation of *mef2ca* mRNA. Loss of Mef2ca function reduced normal muscle growth and diminished the reduction in growth caused by inactivity. We identify eIF4EBP3L as a key regulator of Mef2c translation and protein level following inactivity; blocking eIF4EBP3L function increased Mef2ca translation. Such blockade also prevented the decline in *mef2ca* translation and level of Mef2c and slow myosin heavy chain proteins caused by inactivity. Conversely, overexpression of active eIF4EBP3L mimicked inactivity by decreasing the proportion of *mef2ca* mRNA in polysomes, the levels of Mef2c and slow myosin heavy chain, and myofibril content. Inhibiting the TOR pathway without the increase in eIF4EBP3L had a lesser effect on myofibrilogenesis and muscle size. These findings identify eIF4EBP3L as a key TOR-dependent regulator of muscle fiber size in response to activity. We suggest that by selectively inhibiting translational initiation of *mef2ca* and other mRNAs, eIF4EBP3L reprograms the translational profile of muscle, enabling it to adjust to new environmental conditions.

## Abstract 2

Muscle activity promotes muscle growth through the TOR-4EBP pathway by controlling the translation of specific mRNAs, including Mef2ca, a muscle transcription factor required for normal growth.

## Author Summary

Most genes are transcribed into mRNA and then translated into proteins that function in various cellular processes. Initiation of mRNA translation is thus a fundamental control point in gene expression. Working in a zebrafish model, we have found that muscle activity (or inactivity) can differentially regulate the translation of specific mRNAs and thereby control the growth of skeletal muscle. Emerging evidence suggests that control of translational initiation of particular mRNAs by an intracellular signaling pathway acting through TORC1 is a major regulator of cell growth and function. We show here that muscle activity both activates the TORC1 pathway and suppresses the expression of a downstream TORC1 target—the translational inhibitor eIF4EBP3L. This removes a brake on translation of certain mRNAs. Conversely, we show that muscle inactivity can up-regulate this translational inhibitor, thereby causing reduced translation of these mRNAs. One of the mRNAs targeted in this manner by eIF4EBP3L is Mef2ca, which encodes a transcription factor that promotes assembly of muscle contractile apparatus. Our work thus reveals a mechanism by which muscle growth can be differentially influenced depending on the context of muscle activity (or lack thereof). If this pathway operates in people, it may help explain how exercise regulates muscle growth and performance.

## Introduction

Control of gene function at the level of mRNA translation is emerging as a major regulator of cell and developmental biology, with medical relevance in cancer and elsewhere [1]–[5]. Several broadly acting signaling pathways appear to control translational modules in cultured cells, whereby thousands of mRNAs are coordinately regulated through specific recognition of motifs in the mRNA that remain to be fully defined [6],[7]. One major regulator of such translational modules is the TOR (Target of Rapamycin) pathway [8],[9]. In organisms from yeast to man, TOR regulates protein synthesis, cell size, and general metabolism through several distinct mutliprotein complexes [10]–[12]. A major function of these complexes is to target TOR's protein kinase activity to particular substrates, among them the proteins p70 S6 kinase (p70S6K) and eukaryotic Initiation Factor 4E binding proteins (eIF4EBPs), which regulate protein synthesis [13],[14]. But how TOR achieves specific regulation of diverse cell behaviors in so many different cell types is unclear. Here we define a mechanism that sensitizes specific mRNAs to TORC1 activity in skeletal muscle.

Muscle has advantages for the study of TOR signaling and its role in cell size control. Muscle fibers are among the few cell types that undergo dramatic and reversible changes in size during the normal life of an organism. Moreover, as postmitotic cells, muscle fibers are unaffected by changes in cell cycle that complicate analysis of cell growth and size in proliferative cells [15]. A major regulator of muscle fiber size is exercise, known experimentally as “activity” [16]–[18]. For example, 14 days of mouse hind limb suspension, which decreases electrical activity and the force it elicits, led to reduction of 25%–55% in muscle mass [19],[20]. In humans, reduction of activity leads rapidly to muscle wasting with huge societal and health implications in hospitalized patients and the ageing population. Electrical inactivity has been shown to promote protein breakdown through activation of atrogenes, some of which encode components of both the proteasomal and autophagic degradation pathways [21]–[23]. Inactivity also reduces TORC1 signaling in muscle, which has been suggested to reduce protein synthesis [24],[25] and lead to atrophy.

Zebrafish muscle provides a particularly good system in which to study the effects of activity on muscle growth and in neuromuscular disease [26]–[28]. Two types of muscle fibers are formed in each segmented myotome during the first day postfertilization, a superficial layer of mononucleate slow fibers and a larger number of underlying multinucleate fast fibers [29]–[33]. Over ensuing days, both fiber types undergo significant growth, but the slow fibers remain mononucleate. Zebrafish first move at 17 hours postfertilization (hfp) and undergo repeated contractions during the embryonic and larval stages [34]. Such contractions can readily be blocked without preventing development [35]–[39], providing an opportunity to examine the effect of activity on muscle growth in the developmental context, which is hard to achieve in other vertebrate models.

Here we show that inactivity prevents translation of a specific set of muscle mRNAs, including that encoding Mef2ca, a transcription factor essential for normal muscle growth. Inactivity acts in two ways, first by promoting accumulation of the inhibitor of protein synthesis initiation eIF4EBP3L and second by reducing TORC1 signaling and thus permitting eIF4EBP activity. Active eIF4EBP3L blocks translational initiation of Mef2ca, preventing normal myofibrilogenesis and muscle growth.

## Results

### Muscle Inactivity Reduces Myosin Heavy Chain and Myofibril Width

**Figure 1:**
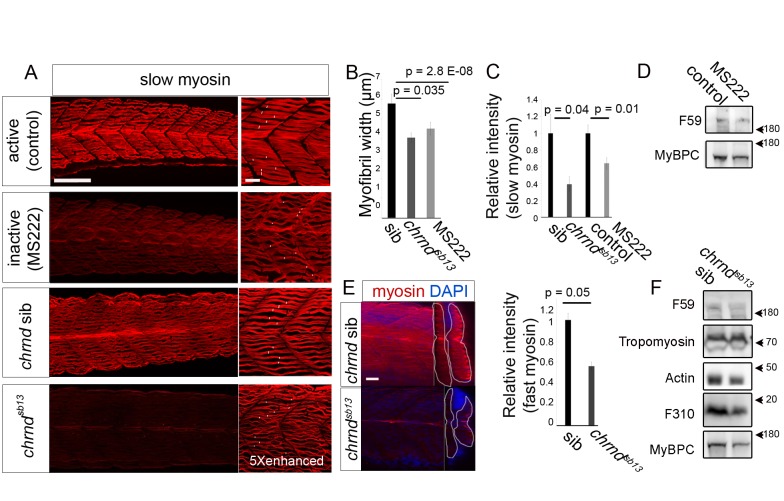
Muscle activity enhances zebrafish myofiber width and myosin level. Muscle activity was blocked either by adding MS222 to the fish water for 24*chrnd* ^sb13/sb13^ mutants lacking the acetylcholine receptor delta subunit, which were identified by their immotility. (A) Confocal stacks of slow MyHC immunostaining in 48 hpf embryos. Note the reduced myofibril content and poor bundling in inactive fish. White bars in the right-hand panels indicate minimal myofibrillar bundle width on each fiber. Fivefold more laser light was used to generate the lower right image. (B) Width of myofibrillar material in well-bundled regions of >5 myofibers in somite 17 were measured from >6 embryos in each condition. (C) Slow MyHC level relative to control in *n* = 15 embryos. (D and F) Western analysis of 48 hpf mutant or MS222-treated embryos, compared to respective controls. (E) Confocal stacks of embryos stained for general MyHC (A4.1025) in lateral (left image) and transverse (right image, somite indicated by white line) view. Graph shows relative MyHC level. *n* = 10 embryos. Bars represent SEM and samples were compared by *t* test. All experiments were repeated at least twice. Scale bar = 90 µm in (A, left), (E), and 23 µm in (A, right).

**Figure 2:**
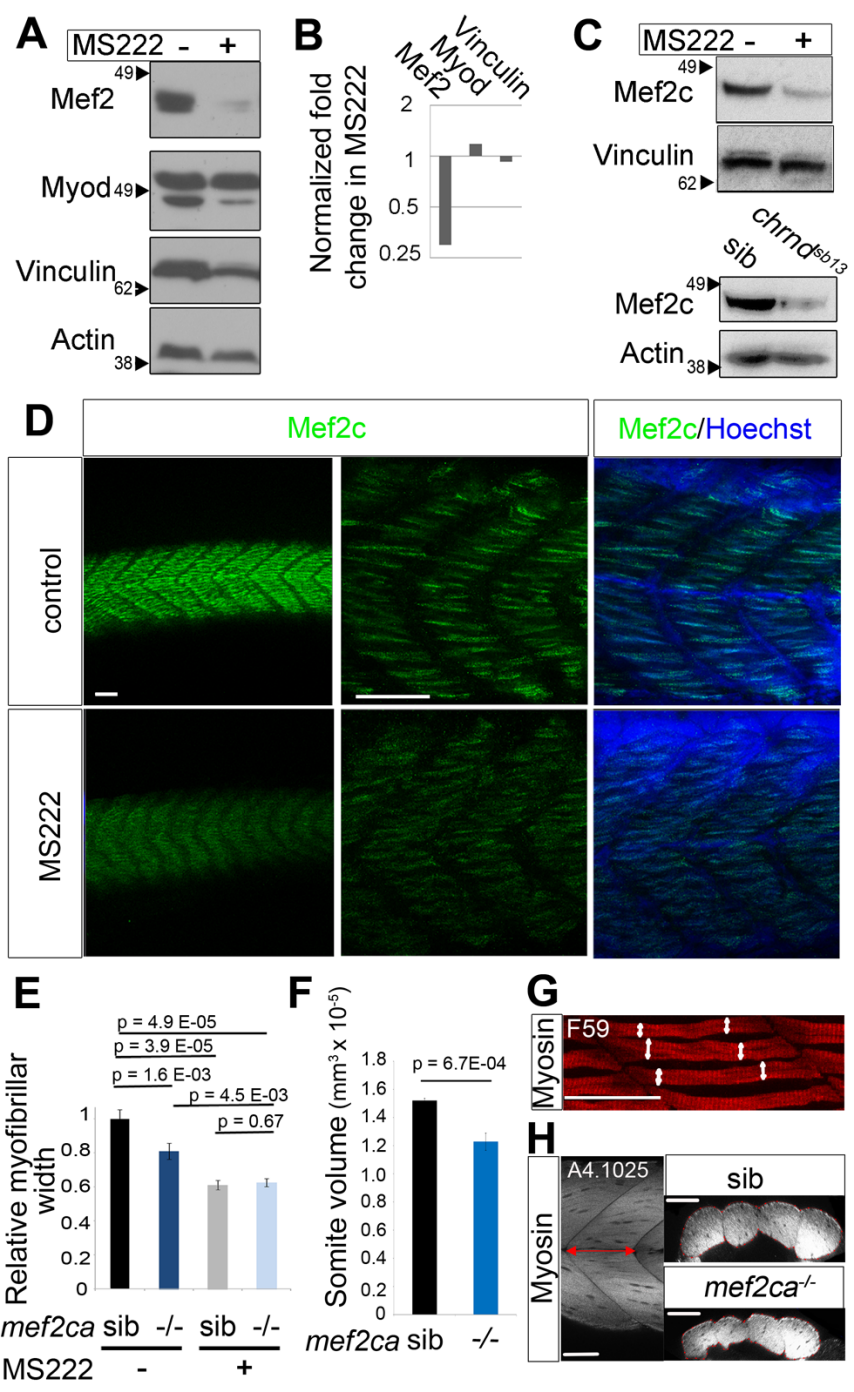
Mef2ca is up-regulated by muscle activity and essential for muscle growth. (A–C) MS222 preferentially suppresses the level of Mef2c protein. Zebrafish embryos were incubated from 31–48 hpf with MS222 and Mef2 and MyoD (A, B) or specifically Mef2c (C) were detected by Western blot (50 embryos/sample). Whereas Mef2 decreased, Myod and Vinculin were unchanged relative to Actin control (B). (D–H) Fibers in the midbody region of wild-type embryos (D) or from a *mef2ca^tn213^* ^/+^ incross (E, F) exposed to MS222 or vehicle for 17 h were immunostained for Mef2c (green) and Hoechst (D) and slow MyHC (E, G) or all MyHC (F, H). Mef2c staining identified mutants at the expected frequency. Myofibril bundle width was determined from at least four fibers in somite 17 of nine embryos in each condition (as shown by white arrows in G). Somite volume was calculated by multiplying the length of the somite 17 by the cross-section area (as shown in red in H, *n* = 17 embryos). Scale bars = 50 µm. Bars represent SEM and samples were compared by *t* test. All experiments were repeated at least twice.

To test the role of activity in zebrafish muscle growth, we examined the reduction in myofiber size in two inactive conditions: the immotile mutant *chrnd* ^sb13/sb13^, which lacks the acetylcholine receptor δ subunit (Figure S1), and after exposure to tricaine mesylate (MS222), a zebrafish anesthetic drug. Both treatments block electrical activity and therefore contraction in muscle. Three parameters were assayed, the width of myofibrillar bundles within slow myofibers, myosin heavy chain (MyHC) immunofluorescence detected with F59 antibody, and the content of various myofibrillar proteins by Western analysis. In the absence of activity, myofibrillar bundle width diminished by 30% in *chrnd^sb13/sb13^* mutants and 22% after MS222 treatment, compared to their respective controls (Figure 1A,B). Similarly, there were decreases in slow MyHC immunofluorescence of 61% and 36% and reductions in slow MyHC by Western of 12% and 52%, respectively (Figure 1A,C,D,F). To investigate whether the decrease in MyHC immunoreactivity was unique to slow fibers or whether it also occurred in fast fibers, we assessed total somite MyHC. MyHC immunoreactivity was decreased by 44% in *chrnd* ^sb13/sb13^ relative to its siblings (Figure 1E). The fast muscle protein myosin light chain, recognized by F310, was reduced by 42%, as were general muscle proteins such as Actin (40%) and MyBPC (35%) (Figures 1D,F and 2A). Thus, in the zebrafish, as in other models, inactivity reduces myofibril content, permitting the use of zebrafish to investigate in vivo the mechanisms that regulate muscle size following disuse.

### Mef2ca Is Up-Regulated by Muscle Activity and Essential for Muscle Growth

We have previously shown that lack of Mef2 activity leads to poor myofibril assembly and that lack of Myod reduces early muscle growth [40],[41]. We therefore tested whether inactivity reduced production of these proteins in parallel with the reduction in muscle growth. MS222 treatment for just 17 h led to a dramatic loss of Mef2 protein without any appreciable change in Myod relative to other control muscle proteins such as Actin and Vinculin (Figure 2A,B). Several Mef2 genes are expressed in differentiated zebrafish muscle, particularly *mef2ca*, *mef2cb*, *mef2d* and *mef2aa* [42]–[44]. Of these, *mef2ca* and *mef2d* are the most abundant at 48 hpf (Figure S2). Mef2C has pleiotropic anabolic effects on murine myogenesis [45],[46]. Consistent with this observation, we found that Mef2c protein was reduced by MS222 treatment and in *chrnd^sb13/sb13^* mutants (Figure 2C). Moreover, whole-mount immunofluorescence confirmed a decrease of Mef2c in myotome nuclei following MS222 treatment (Figure 2D).

To test whether a decrease in Mef2c contributes to the reduction in myofiber width, mutant and sibling embryos from a *mef2ca^tn213/+^* incross were compared. A 20% decrease in myofibril bundle width and in somite volume was observed in 48 hpf (Figure 2E,G) and 5 dpf mutants (Figure 2F,H). These findings show that Mef2ca activity is required for normal muscle growth.

We next asked whether Mef2c mediates the effect of muscle activity on myofiber width. MS222 causes an even greater reduction of myofibril bundle width than a complete loss of Mef2ca, indicating that muscle activity does more than promote Mef2ca activity (Figure 2E,G). Nevertheless, in the absence of activity, loss of Mef2ca has no effect on myofibril bundle width (Figure 2E,G), suggesting that the Mef2ca pathway is inactive when muscle itself is inactive. These results argue that Mef2ca contributes to muscle activity-induced growth, but is not the sole mechanism.

### Muscle Activity Regulates the Translation of *mef2ca*

**Figure 3:**
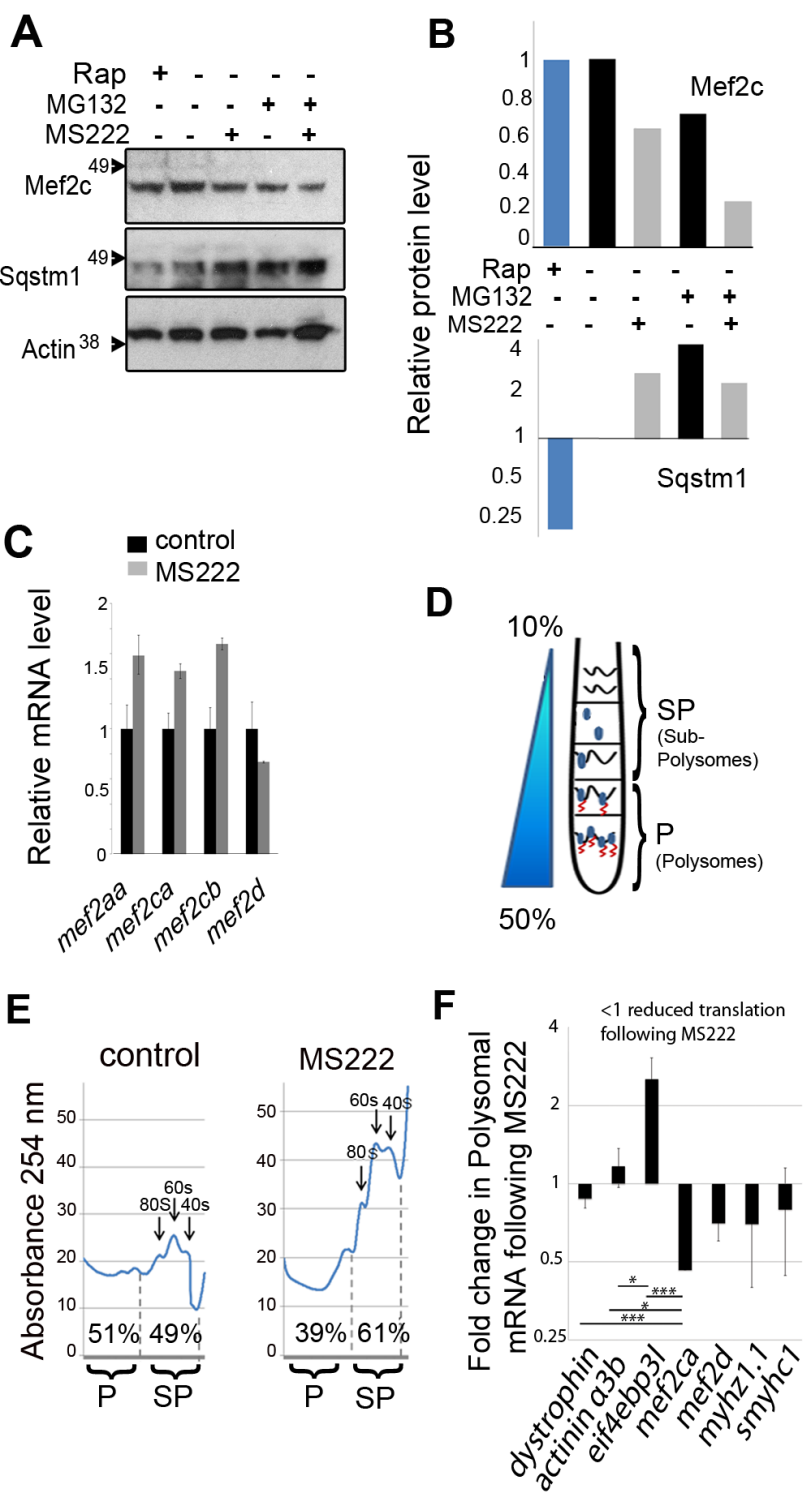
Muscle activity regulates translation of *mef2ca* mRNA. Zebrafish embryos were exposed to MS222, Rapamycin [34], or vehicle control. (A and B) Muscle activity does not regulate Mef2 protein via the proteasomal or autophagic pathways. (A) Western blot of whole embryos (48 hpf; 50/sample) incubated with 100 nM MG132 or DMSO vehicle for 1 h prior to and then during 17 h MS222 treatment. (B) Quantification of Mef2c and full-length 50 kd Sqstm1 bands relative to Actin. (C) qPCR of the indicated mRNAs relative to *actin* following MS222 or vehicle (48 hpf 20 embryos/sample). (D) Schematic of separation of cytoplasmic extract on a sucrose gradient into light sub-polysome (SP) and heavy polysome (P) fractions to reveal fraction of mRNA in each. (E) Polysomal profiles of nucleic acid from the gradient of active control and inactive MS222 zebrafish. Peaks indicate 40 s, 60 s subunits, and 80 s monosomes. The amount of nucleic acid in the polysome (P) and subpolysome (SP) fractions were calculated from the areas between the dashed lines. (F) Differential regulation of muscle mRNAs by activity. Proportion of each specific mRNA in the P fraction, SP fraction, and unfractionated (total) was measured by qPCR. Activity-dependent change in translation rate at 48 hpf was determined as the ratio of polysomal/total in MS222 to polysome/total in control (70 embryos/sample). The level of each gene in the fraction was normalized to its level in the total to rule out change in transcription. To present the average from several experiments, the translation change was normalized to the relative change in *mef2ca*. Bars represent SEM (*n* = 4) and samples were compared by *t* test. All experiments were repeated at least twice.

The preferential reduction in Mef2c protein in inactive muscle could stem from either increased proteolysis or reduced synthesis. In adult muscle, inactivity potently activates both the proteasomal and autophagy-lysosomal proteolysis pathways [47],[48]. To test the role of proteasomal degradation in Mef2 regulation, muscle activity was abolished in the presence of MG132, an inhibitor of the proteasome. MG132 treatment of fish embryos up-regulated known targets of the proteasome, p53 and Sqstm1 [49],[50], providing positive controls for MG132 efficacy (Figures 3A and S3A). Inactivity led to decrease in Mef2 levels even in the presence of MG132 (Figure 3A,B), indicating that Mef2c decline is not due to proteasomal degradation. Congruently, rather than increasing Mef2c, MG132 alone decreased Mef2c, suggesting that proteasomal degradation is not a significant Mef2c turnover pathway in embryonic muscle (Figure 3A,B).

Autophagy was then assessed by the level of Sqstm1, which is a target to autophagic degradation and marker of autophagic flux [51]. The TORC1 inhibitor rapamycin, a known and potent inducer of autophagy, reduced zebrafish full-length 50 kd Sqstm1, serving as a positive control by showing that autophagy reduces Sqstm1 in zebrafish (Figure 3A,B). The level of zebrafish Sqstm1 was not decreased by muscle inactivity (Figure 3A,B). As a further control, we found no increase in *sqstm1* mRNA that could have compensated for a loss of Sqstm1 protein ([52]; Figure S3B). Therefore, muscle inactivity did not induce autophagy in embryonic zebrafish. Moreover, inhibition of autophagy with the lysosomal inhibitor chloroquine did not block the reduction in Mef2c protein caused by inactivity (Figure S3C). Furthermore, Mef2c level appeared unchanged under rapamycin-induced autophagy, indicating that Mef2c is not a target for autophagic degradation (Figure 3A,B). Thus, the reduction of Mef2c in inactive muscle was apparently not due to enhanced degradation by the ubiquitin-proteasome or autophagy pathways, which suggested that its synthesis is regulated.

To determine whether the reduction in Mef2 was due to a reduction in mRNA synthesis, we used qPCR. Upon MS222 exposure, no decrease in mRNA level for any of the four Mef2 genes was detected (Figure 3C). Thus, reduced mRNA levels does not account for loss of Mef2 in inactive muscle.

The lack of demonstrable change in Mef2 mRNAs or Mef2c proteolysis in inactive embryonic muscle prompted us to examine changes in protein synthesis, particularly because muscle activity is known to promote protein synthesis [53],[54]. We therefore developed a method to quantify RNA associated with polysomes in fish embryos. Polysomes are complexes that contain actively translating mRNA associated with two or more ribosomes and represent the densest RNA-containing fraction of cytoplasm. Embryo cytoplasmic lysate was subjected to sucrose density gradient separation and the location of the zebrafish polysome-containing fraction determined (Figures 3D,E and S4). As controls, polysomes were diminished by addition of EDTA to the extract, which dissociates polysomes, and enhanced by addition of cycloheximide, which stabilizes polysomes by inhibiting translational elongation, thereby stalling ribosomes on the mRNA (Figure S4). The effect of activity on polysomes was then determined. Following 17 h MS222 exposure, the amount of nucleic acid in the polysome fraction was reduced compared to that in the 80S monosome and 60S and 40S ribosomal subunit peaks (Figure 3E). Polysome-associated RNA decreased by ∼10% in MS222 compared to active controls. This finding shows that muscle inactivity reduced protein synthesis in zebrafish, providing a potential explanation for the reduction in Mef2c in inactive muscle.

To determine whether specific mRNAs such as those encoding Mef2 are subject to translational regulation, the proportion of *mef2ca* mRNA in polysomes was assessed. mRNA from the polysome (heavy fraction; P) was purified and the change in mRNAs following MS222 treatment determined by qPCR (Figure 3F). Translation of *mef2ca* reduced by 55% in the absence of activity, whereas translation of various controls, such as *dmd* (*dystrophin*) and *actn3b* (*α-actinin 3b*), differed significantly, showing no reduction (Figure 3F). We did not detect significant reduction in translation of other mRNAs tested by qPCR. As expected, relative reduction of mRNAs in the polysomal fraction was complemented by a relative increase in the subpolysomal fraction (Table S2). These findings show that there is preferential loss of specific mRNAs from polysomes in inactive muscle, and that Mef2ca protein translation is specifically promoted by muscle activity.

### Muscle Inactivity Down-Regulates the TORC1 Pathway

**Figure 4:**
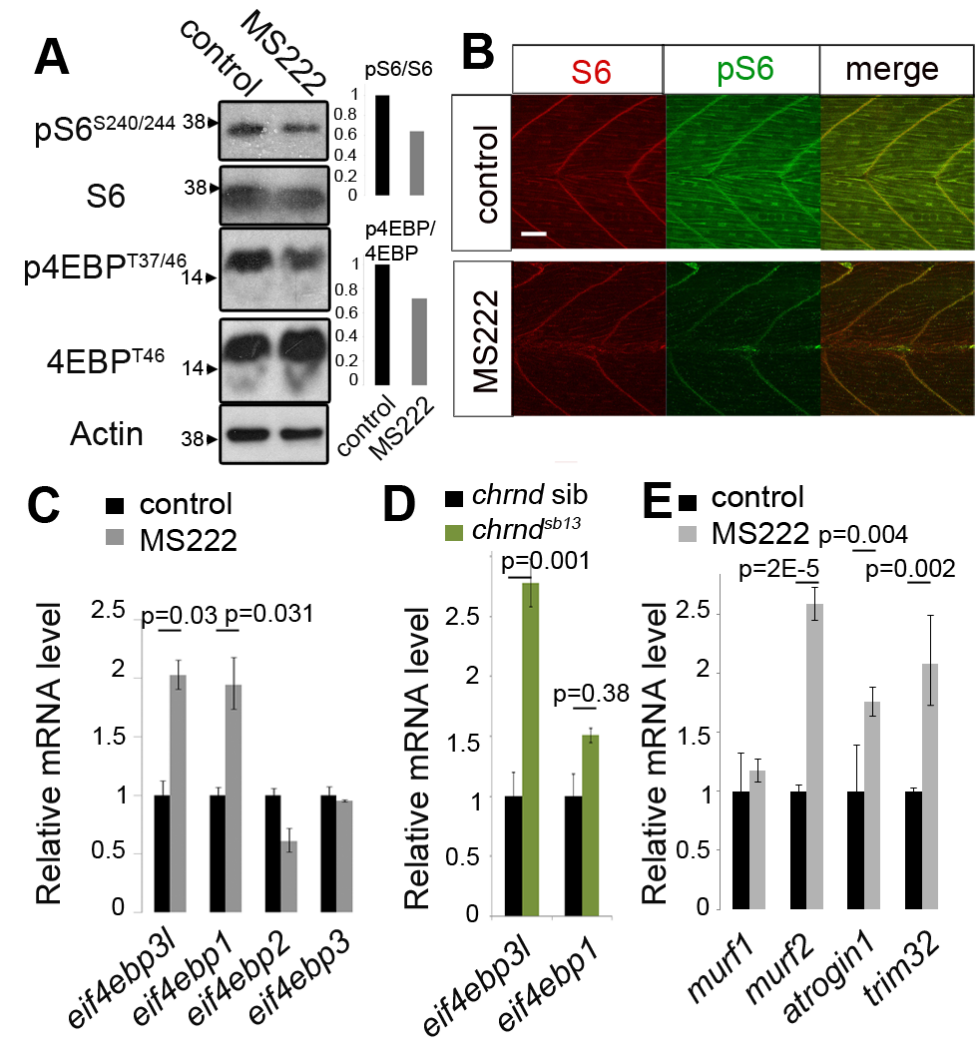
Muscle inactivity down-regulates the TOR pathway and induces eIF4EBP3L mRNA. Wild-type (A–C, E) or *chrnd^sb13/+^* incross zebrafish embryos were incubated with (grey bars) or without (black bars) MS222 for 17–24 h. At 48 hpf, embryos were analyzed by Western analysis (A), immunostaining (B), or qPCR relative to *actin* (20 embryos/sample; C). (A and B) Muscle inactivity reduces TORC1 activity. Phosphorylation levels of downstream TORC1 targets were reduced both in whole embryo (A) and in muscle tissue (B). Scale = 200 µm. Note that both eIF4EBP antisera likely detect eIF4EBP1, 2, and 3L as the epitope sequence is conserved between human and zebrafish (see Figure S4C). (C and E) Electrical inactivity specifically increased *eif4ebp3l* and *eif4ebp1* mRNA in whole embryos (C). Several zebrafish E3 ligase atrogenes showed similar increases in mRNA level (E). (D) Specific loss of muscle activity in acetylcholine receptor δ mutants (identified by their immotility) induces *eif4ebp3l*, but not *eif4ebp1* mRNA, compared to siblings. Actin served as a control (A, C–E) for normalization. Error bars represent SEM and samples were compared by *t* test. All experiments were repeated at least twice.

TORC1 was previously shown to differentially regulate translation [1],[2],[55],[56], and is a major player in activity-related muscle growth [18],[19]. We therefore asked whether muscle activity affects TORC1 activity in the embryonic zebrafish. TORC1 pathway activity was assessed by examining the phosphorylation of eIF4EBP and ribosomal protein S6, known downstream targets of the TORC1 complex involved in translational regulation [57]. When zebrafish embryos were exposed to MS222, Western analysis revealed that phosphorylation of S6^S240/244^ and eIF4EBP^T37/46^ was decreased by 40% and 30%, respectively, compared to untreated controls (Figure 4A). As these proteins are widely expressed, immunostaining of phopho-S6 (pS6) and total S6 was used to assess changes occurring in muscle tissue. MS222 caused a decrease in muscle pS6 compared to controls (Figure 4B). The levels of S6 itself also appeared somewhat down-regulated, although no loss of ribosomal 18S or 28S rRNA was detected in inactive embryos (Figure S5). Thus, muscle activity activates the TORC1 pathway, making it a good candidate as a regulator of the translational response to activity.

TORC1 is inhibited by rapamycin, which is known to reduce muscle growth [18],[19]. Rapamycin treatment of zebrafish embryos reduced muscle growth, but to a lesser extent than inactivity (Figure S6A–C). Strikingly, although rapamycin reduced muscle growth, it was much less effective than inactivity at reducing Mef2c (Figure 3A,B; see also [58]) or Myosin and Actin protein accumulation (Figure S6D). Congruently, rapamycin was more effective at reducing S6 phosphorylation than eIF4EBP phosphorylation (Figure S7), whereas inactivity appeared equally effective on each TORC1 target (Figure 4A). Thus, the greater effectiveness of inactivity compared to rapamycin on reducing muscle growth correlates with the ability of inactivity to down-regulate Mef2c translation.

TORC1 can affect both global translation by promoting rRNA synthesis via S6K [59],[60], and differential translation by blocking eIF4EBPs [1],[2]. eIF4EBPs prevent translational initiation by binding eIF4E, inhibiting its interaction with eIF4G, and thereby preventing recruitment of the 40S ribosomal subunit to the mRNA [61],[62]. To determine which TOR-dependent translational regulation operates in inactive muscle, we first looked at rRNA. We tested the levels of rS28 and rS18; after 16 h MS222 they were not reduced (Figure S5). On the other hand, reduction in protein level following inactivity occurred rapidly and varied from protein to protein (Figures 1, 2, and 3), which implied that TORC1-dependent differential translational regulation occurs, potentially via eIF4EBP.

### Muscle eIF4EBP3L Is Regulated by Activity

Active TORC1 phosphorylates and inhibits eIF4EBP, thereby promoting translational initiation of specific mRNAs [1],[2],[63],[64]. The vertebrate eIF4EBP protein family consists of three members—eIF4EBP1, eIF4EBP2, and eIF4EBP3—that share >55% amino acid identity (Figure S8A). Each harbors a conserved eIF4E binding site, YDRKFLL, two canonical TOR phosphorylation sites, TPGGT, and several transregulatory phosphorylation sites (Figure S8C). Zebrafish have four eIF4EBPs, with an eIF4EBP3 duplicate named eIF4EBP3-like (eIF4EBP3L; Figure S8B). eIF4EBP3L has greater homology (78% identity) to human eIF4EBP3 than between any other zebrafish∶human eIF4EBP pair (Figure S8B). Two eIF4EBP3s are found in many teleosts (www.ensembl.org), suggesting they arose from the early teleost genome duplication. The zebrafish eIF4EBP genes have distinct tissue expression: *eif4ebp1* and *eif4ebp2* widely and highly in head and neural tissue, *eif4ebp3* only abundant in pancreas, and *eif4ebp3l* in the somite, eye, and branchial arch region [65]. Thus, *eif4ebp3l* appeared to have a particular role in muscle tissue.

The effect of muscle activity on eIF4EBP gene expression was assessed. Upon exposure to MS222, the level of *eif4ebp3l* mRNA increased 2-fold, particularly in muscle, but did not otherwise change tissue mRNA distribution. MS222 also induced *eif4ebp1* mRNA 1.9-fold (Figures 4C and S8D). To rule out effects of MS222 on neural activity and to examine the effect of activity specifically in muscle tissue, we assessed mRNA levels in the *chrnd^sb13^* mutant, in which muscle alone is inactive, and found a 2.5-fold increase in *eif4ebp3l* mRNA but without change in *eif4ebp1* mRNA (Figure 4D). These data indicate a specific role for *eif4ebp3l*, and not *eif4ebp1*, in the response to muscle inactivity.

Separately, we analyzed the effect of inactivity on so-called atrogenes, genes known to be induced in muscle atrophy caused by malnutrition, denervation, sepsis, or ageing in adult mammalian muscle [66],[67]. We found, as in the case of *eif4ebp3l*, a 2-fold increase in *atrogin-1*, *murf2*, and *trim32* mRNAs induced by MS222 (Figure 4E) and similar induction in *chrnd^sb13^* mutants (Figure S9). These results show that inactivity up-regulates a suite of mRNAs regulating protein turnover in muscle, including *eif4ebp3l*.

When translation of *eif4ebp3l* mRNA was analyzed by polysomal fractionation, a significantly increased fraction was in polysomes (Figure 3E). These increases in both *eif4ebp3l* mRNA level and its translation rate suggest that eIF4EBP3L protein will increase in inactive muscle. Lacking an eIF4EBP3L-specific antibody, we could not show such up-regulation directly, but total eIF4EBP protein did appear higher in inactive embryos (Figure 4A). We hypothesized that increased eIF4EBP3L might cause certain mRNAs to become more sensitive to TORC1-regulation in inactive muscle. Consistent with this idea, more significant reduction in myofibrilogenesis was caused by MS222 than by inhibiting TORC1 with rapamycin alone (Figure S6). Thus, muscle inactivity both increased eIF4EBP level and reduced eIF4EBP phosphorylation, raising the possibility that eIF4EBP3L drives the differential translational repression in inactive muscle.

### Active eIF4EBP3L Suppresses Mef2ca Translation

**Figure 5:**
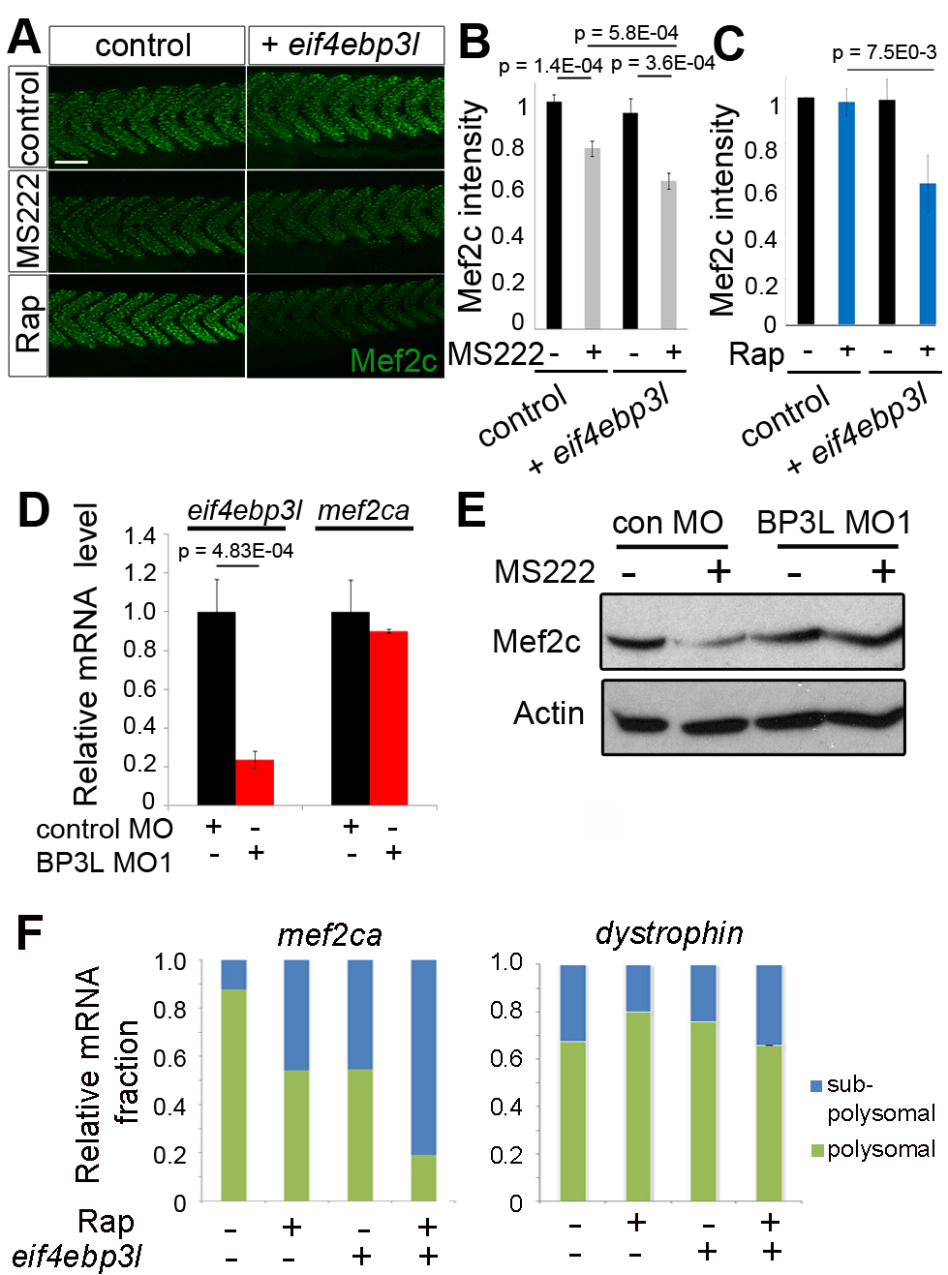
Overexpression of eIF4EBP3L reduces and knockdown rescues Mef2c. Zebrafish embryos injected with RNA encoding eIF4EBP3L (A–C, F) or control MO or BP3L MO1 (D, E) were grown to 48–54 hpf and some exposed to MS222 for the last 17 h (A, B, E) or to 5 µM rapamycin for 6 h (Rap; A, C, F) or remained as untreated controls. (A–C) Mef2c immunostaining intensity quantified by confocal microscopy in 54 hpf embryos shows that overexpression of eIF4EBP3L reduced Mef2c protein in inactive (B) or rapamycin-treated (C) muscle. *n* = 60 (B) and *n* = 20 (C) embryos/sample. Scale bar = 90 µm. (D) Level of indicated mRNAs assayed by qPCR shows that BP3L morphants have reduced *eif4ebp3l* mRNA. (E) Western analysis shows that BP3L MO1 prevented decrease in Mef2c caused by inactivity compared to actin loading control. Bars represent SEM. Samples were compared by *t* test. All experiments were repeated at least twice. (F) Polysomal fractionation followed by triplicate qPCR for *mef2ca* and *dmd* mRNAs on polysomal and subpolysomal fractions.

As inactivity increased active (unphosphorylated) eIF4EBP3L and reduced translation of *mef2ca* mRNA and myogenesis, we tested whether Mef2ca protein is down-regulated when eIF4EBP3L is active. Embryos were injected with mRNA encoding eIF4EBP3L to achieve a 2-fold increase, comparable to that induced by inactivity (Figure S10A). First, the intensity of Mef2c immunofluorescence was measured in confocal stacks of skeletal muscle nuclei (Figure 5A). Overexpression of eIF4EBP3L in active control muscle did not significantly affect Mef2c levels, in contrast to the reduction observed with MS222 (Figure 5A,B). However, overexpression of eIF4EBP3L in inactive MS222-treated embryos, a condition in which eIF4EBP is hypophosphorylated (Figure 4A), significantly decreased Mef2ca protein levels by a further 20% below that caused by MS222 alone (Figure 5B). We conclude that eIF4EBP3L can inhibit Mef2c accumulation in muscle, but that muscle activity normally suppresses eIF4EBP3L activity.

To determine whether inhibition of TORC1 activity can mimic the effect of MS222 on eIF4EBP3L, rapamycin was applied to embryos injected with *eif4ebp3l* mRNA and controls. Rapamycin suppressed TORC1 activity in zebrafish embryos, reducing both phosphoS6 and phospho4EBP (Figure S7). As in Figure 3A, rapamycin had no effect on Mef2c level in mock-injected control active muscle (Figure 5A,C), presumably because the low endogenous eIF4EBP3L level was insufficient to cause a significant reduction in initiation of Mef2ca translation. However, when eIF4BP3L was overexpressed, rapamycin caused a 40% reduction in Mef2c level (Figure 5C), comparable to that triggered by MS222 (Figure 5B). Thus, TORC1 activity suppresses eIF4EBP3L function in active skeletal muscle.

To test the role of endogenous eIF4EBP3L, we knocked down eIF4EBP3L by injecting 1–2 cell stage embryos with morpholino antisense oligonucleotides (MO) targeting the exon1-intron1 splicing site (BP3LMO1) and against the start codon (BP3LMO2) of *eif4ebp3l*. In the absence of specific antibody against eIF4EBP3L, we verified the efficacy of BP3LMO1 by qPCR and found a decrease of 80% in *eif4ebp3l* mRNA level, whereas *mef2ca* mRNA was not affected, relative to *actin* (Figure 5D). In embryos injected with control MO, inactivity led to a 40% decrease in Mef2c protein. This decrease was prevented when eIF4EBP3L was knocked down (Figure 5E). Thus, eIF4EBP3L is necessary for the reduction in Mef2c in inactive muscle.

The involvement of eIF4EBP3L in Mef2c reduction caused by inactivity is most simply explained by altered translational initiation. To test this hypothesis, embryos injected with *eif4ebp3l* RNA were subjected to polysomal fractionation. Overexpression of eIF4EBP3L led to a 40% decrease in *mef2ca* mRNA in the polysome fraction, whereas *dystrophin* mRNA, a negative control that was unaffected by activity (Figure 3F), showed no change (Figure 5F). Moreover, addition of rapamycin to inhibit TORC1 enhanced the effect of eIF4EBP3L overexpression (Figure 5F), and decreased Mef2c protein (Figure 5C). Whereas either *eif4ebp3l* RNA injection or 6 h rapamycin treatment alone reduced *mef2ca* mRNA in polysomes, neither was sufficient to lower Mef2c protein (Figure 5A,C,F). This finding suggested that, in order for the translation inhibition to lead to reduction of Mef2c protein level, such inhibition must exceed a threshold, as occurs when both eIF4EBP3L is up-regulated and TORC1 is inhibited.

To confirm that endogenous eIF4EBP3L specifically regulates *mef2ca* translation, we reduced eIF4EBP3L level with morpholino and performed polysome fractionation on active and inactive muscle. In the presence of control MO, muscle inactivity reduced *mef2ca* translation by 37% (*p* = 0.0035, while having no effect on *dmd* mRNA; Figure S11). In contrast, in the presence of eIF4EBP3L MO, muscle inactivity had no significant effect on *mef2ca* translation (Figure S11). In addition, knockdown of eIF4EBP3L in both active and inactive muscle led to an increase in *mef2ca* mRNA in polysomes, but did not affect *dmd* mRNA (Figure S11). Nevertheless, Mef2ca protein did not accumulate upon eIF4EBP3L knockdown (Figure 5E). These data suggest that eIF4EBP3L is required for the reduction in *mef2ca* translation in inactive muscle and retains the ability to target specific mRNAs in active muscle.

### eIF4EBP3L Activity Blocks Normal Myofibrilogenesis

**Figure 6:**
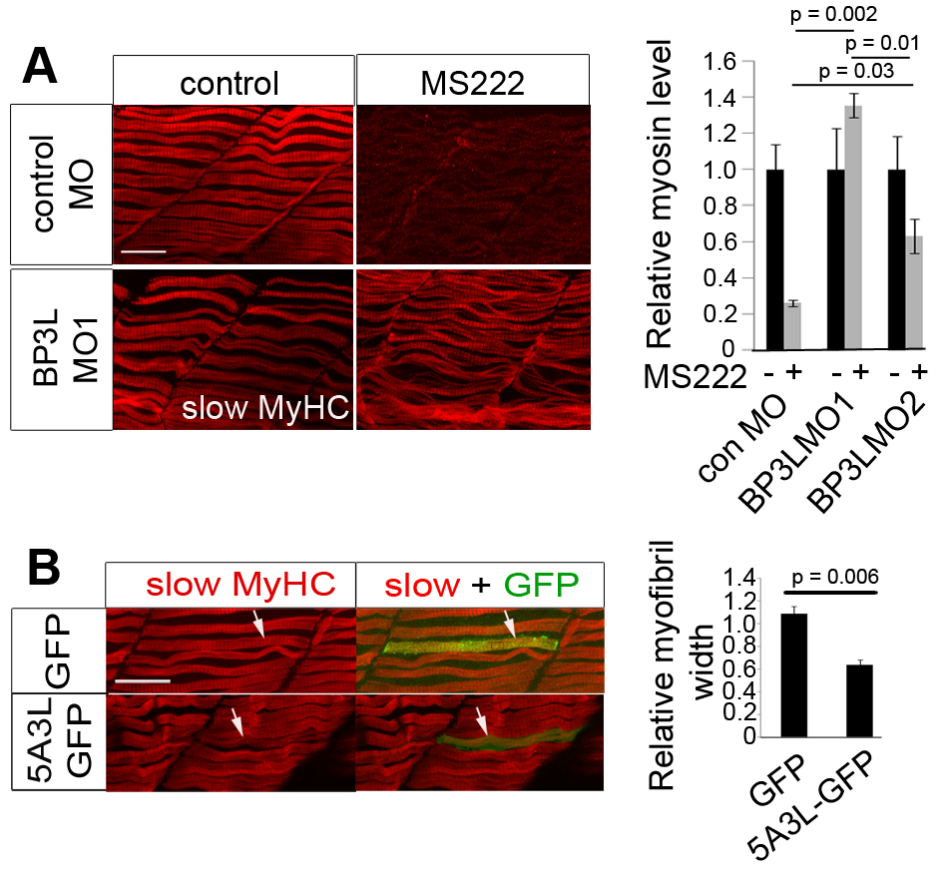
Active eIF4EBP3L regulates myofibrilogenesis following inactivity. Embryos were injected with the indicated MO, some exposed to MS222 for the last 17(A) or with plasmid DNA encoding a constitutively active eIF4EBP3L and GFP (5A3L-GFP) or empty vector (GFP) (B) were grown to 48–54 hpf and immunostained for slow MyHC. (A) BP3L MO1 prevents, and BP3L MO2 reduces, the decrease in slow MyHC caused by inactivity. Immunostaining (left panels) was quantified by confocal microscopy (right panel; *n* = 15 embryos/sample). (B) Overexpression of 5A3L reduces myofibril bundle width. Following heat shock, the size of slow myofibril bundles in GFP+ fibers was determined relative to their GFP− neighbors (*n* = 9 embryos/sample). Bars represent SEM and samples were compared by *t* test. All experiments were repeated at least twice. Scale bar = 23 µm.

As Mef2ca regulates myofibrilogenesis and fiber growth (Figure 2E,F), we asked whether eIF4EBP3L also does so. In active control muscle, *eif4ebp3l* morphant myofibrilogenesis appeared similar to that in controls (Figure 6A). Inactivity caused a 75% decrease in slow MyHC signal in embryos injected with control MO. However, this decline was prevented when eIF4EBP3L was knocked down with either BP3L MO (Figure 6A). We conclude that eIF4EBP3L prevents normal myogenesis under inactive conditions.

Inactive muscle has both increased *eif4ebp3l* mRNA and decreased phosphorylation of TORC1 targets, both of which cooperate to specifically suppress translation (Figures 4 and 5). To test whether eIF4EBP3L can influence myofibrilogenesis independent of TORC1 activity, we generated constitutively active eIF4EBP3L by mutating the five potential threonine/serine phosphorylation sites to alanine, thereby generating 5A3L (Figure S8C). However, injecting 5A3L RNA led to early embryonic death, possibly due to widespread translational repression.

To overcome the early lethality, we cloned 5A3L into a zebrafish heat-shock–inducible expression vector also containing a GFP marker to achieve controlled mosaic expression in muscle. Slow myofibrilogenesis was assayed at 54 hpf by comparing GFP-expressing fibers to unmarked neighboring fibers within the same somite. Overexpression of 5A3L led to a 36% decrease in myofibril bundle width (Figure 6B), whereas controls expressing GFP alone showed no significant change. Moreover, a variety of other cell types in epidermis, notochord, and neural tube showed no detectable morphological change upon 5A3L overexpression (Figure S10B). Thus, active eIF4EBP3L reduces slow myofibrilogenesis.

## Discussion

The current work provides three new insights into translational control in the context of skeletal muscle tissue in vivo: first, that muscle activity differentially regulates translation of specific mRNAs; second, that the conserved protein eIF4EBP3L, a downstream target of TORC1 signaling, regulates translation and myofibrilogenesis in response to activity; and third, that Mef2ca is among the translationally controlled targets that mediate the effect of electrical activity on myofibrilogenesis and thereby muscle growth.

### Differential Translational Control in Response to Altered Muscle Activity

**Figure 7:**
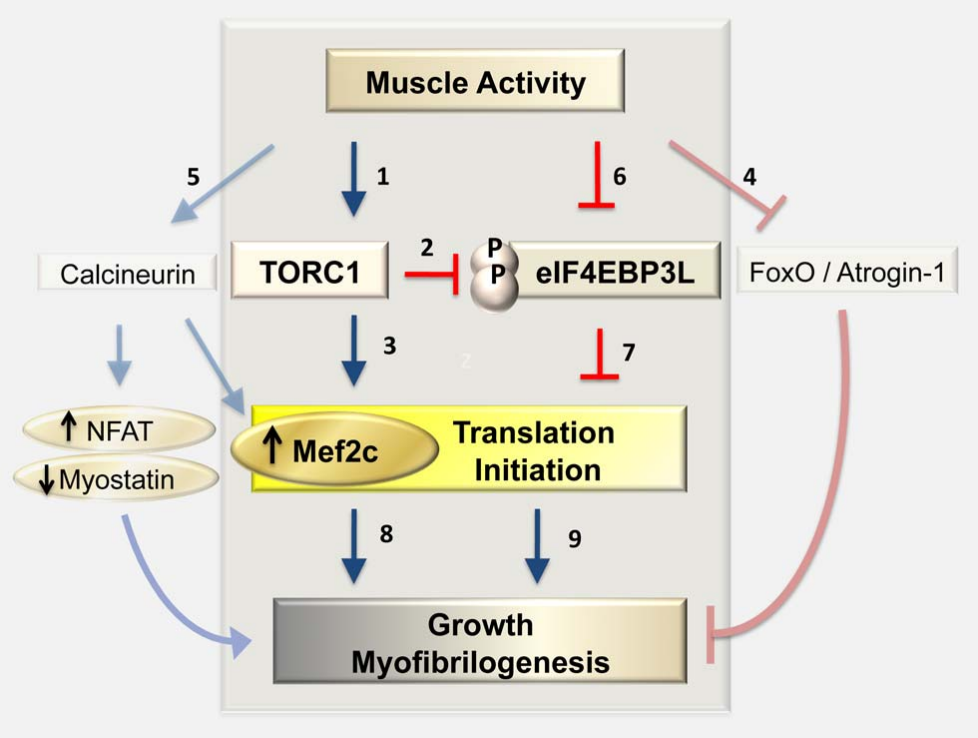
Model of how muscle activity stimulates muscle growth through differential translational regulation. Previous studies indicated a role of TORC1 (a known cell size regulator) in activity-dependent muscle growth (1). TORC1 regulates growth by inhibiting the translation initiation inhibitor eIF4EBP (2) and by activation of S6K (3) [108], both leading to increased translation. In the absence of muscle activity, muscle fibers undergo atrophy, a reduction in size and strength. Both the FoxO/Atrogin-1 axis (4) and the calcineurin pathway (5) contribute to atrophy. We found that during muscle development lack of muscle activity increases the levels of eIF4EBP3L in muscle (6) and reduces the activity of TORC1 pathway (1), preventing eIF4EBP3L phosphorylation (2) and thereby activating it. High levels of active eIF4EBP3L prevent initiation of protein synthesis from specific mRNAs (7). Among mRNAs regulated by TORC1/eIF4EBP3L is that encoding the transcription factor Mef2ca, which is also regulated by calcineurin and is required for normal myofibrilogenesis and muscle growth (8). We hypothesize that other mRNAs are also differentially regulated by the activity/TORC1/eIF4EBP3L axis (9) and contribute to muscle homeostasis along with the FoxO and calcineurin pathways.

By applying polysomal mRNA fractionation to whole zebrafish embryos, we have developed a method to analyze changes in their translational control. We find that muscle activity promotes translation of a specific mRNA that is normally expressed in muscle and encodes an important muscle regulator, namely Mef2ca (Figure 7). The translational changes we highlight occur in relation both to total RNA content of the embryo and to other muscle-specific mRNAs, such as Dystrophin and α-Actinin-3b. The reduced translation triggered by inactivity leads to rapid loss of Mef2 protein.

Muscle electrical activity controls many aspects of adult muscle character, including contractile and metabolic properties and fiber size. In developing amniote primary muscle, however, the major effect of reduced activity is reduced growth rate [68],[69] (reviewed in [70]). We show here that growth is also reduced in electrically inactive zebrafish muscle, consistent with previous comments [39]. Without activity, initial differentiation of slow and fast muscle fibers appears normal, but accumulation of myofibrillar protein is diminished. In the mononucleate slow fibers, which form in normal numbers, myosin fails to assemble into myofibrils at the normal rate, leading to a reduced width of myofibril bundles.

The pathways that mediate the effects of activity on muscle growth are many and fall into two broad categories, catabolic pathways triggered by inactivity and anabolic pathways promoted by activity. The relative contribution of proteolysis or protein synthesis changes to muscle mass decrease in the inactive condition is a controversial topic. Although catabolism is a major driver, altered protein synthesis may also contribute [54],[71],[72]. Our data reveal eIF4EBP3L as a new regulator of muscle growth that is up-regulated in inactive muscle and inhibits translational initiation through targeting a specific subset of mRNAs. A high-throughput approach will be needed to define the full set of mRNAs showing altered translation in inactive muscle. We also found that muscle inactivity causes a 2-fold increase in E3 ligase atrogenes, known targets of FoxO, that trigger proteasomal degradation of muscle constituents [73],[74]. Thus, our data suggest that both decreased protein synthesis and increased catabolism are triggered in response to inactivity in developing muscle.

How does inactivity up-regulate *eif4ebp3l* expression? In other systems, eIF4EBP mRNAs are up-regulated by the FoxO pathway [75]–[79]. Considering the increase in transcription of both *eif4ebp3l* and known atrogenes in inactive muscle, we hypothesize that FoxOs are activity-dependent regulators of transcription of both *eif4ebp3l* and the E3 ligases in growing zebrafish muscle.

We show that loss of eIF4EBP3L function prevents atrophic changes caused by inactivity during growth. If a human eIF4EBP functions similarly in adults, it could constitute a novel therapeutic target for prevention of acute muscle wasting. Changes in other eIF4EBPs, such as eIF4EBP1, have been observed in muscle atrophy caused by fasting, and have been suggested to regulate general translation rate [80]. Interestingly, we observed significant increase in *eif4ebp1* mRNA in our zebrafish inactivity model only when whole body electrical activity, not just muscle activity, was blocked. As *eif4ebp1* is highly expressed in the central nervous system, these data suggest that eIF4EBP1 may respond to activity by mediating growth-related changes, such as synaptic elaboration, during neural development [81],[82].

### eIF4BP3L as a Gatekeeper of TORC1 Differential Translational and Growth

eIF4EBPs block translation by binding to eIF4E and preventing eIF4G attachment to the 5′ cap-dependent mRNA initiation complex. TORC1 pathway activity phosphorylates eIF4EBP, inhibiting its interaction with eIF4E and thereby promoting translational initiation. The TORC1-4EBP pathway was previously thought of as a general regulator of 5′ cap-dependent translation [61],[63],[83]. However, recent large-scale screens revealed differential mRNA translation by TORC1-4EBP in cultured cells. Specific sequences within an mRNA, known as TOP-like or Pyrimidine-Rich Translational Elements (PRTEs), correlate with sensitivity to translational regulation [1],[2]. We find similar differential control by TORC1-4EBP3L in zebrafish muscle in vivo. The *mef2ca* mRNA that is translationally repressed by eIF4EBP3L has a PRTE in the 5′ UTR, as does *mef2d* (Table S3). However, as *eif4ebp3l* itself also has a PRTE, defining an mRNA sequence consensus for eIF4EBP3L translational repression in muscle will require further analyses.

Muscle inactivity reduced phosphorylation of eIF4EBP and ribosomal protein S6, suggesting that loss of TORC1 pathway activity triggered the translational and growth reduction. However, in zebrafish, inactivity reduces Mef2c levels and muscle growth more effectively than the TORC1 inhibitor rapamycin, as previously observed in mammals [19]. We found that up-regulation of eIF4EBP3L in inactive muscle helps explain why inactivity is more effective than rapamcyin alone. In active muscle, eIF4EBP3L levels are low and the activity of eIF4EBP3L is further suppressed by phosphorylation by TORC1. In contrast, when eIF4EBP3L is induced in inactive muscle, translation becomes more sensitive to TORC1 activity. In this situation, the inactivity also lowers eIF4EBP3L phosphorylation, leading to a high level of active eIF4EBP3L, which specifically inhibits translation of certain mRNA targets, such as *mef2ca*. We confirm this hypothesis by showing that when eIF4EBP3L is overexpressed, Mef2c level becomes more sensitive to rapamycin. Thus, the level of eIF4EBP3L acts as a gatekeeper, controlling the sensitivity of muscle to TORC1 activity.

In contrast to eIF4EBP1 and eIF4EBP2, the function of eIF4EBP3 has been unclear [84],[85]. We show that eIF4EBP3L blocks translational initiation of specific mRNAs and simultaneously sensitizes the inactive muscle to TORC1. One advantage of such control is that, when activity returns, protein synthesis can rapidly resume as soon as TORC1 activity increases and phosphorylates eIF4EBP3L. Thus, if the pathway we have revealed in developing muscle were to also operate in inactive adult muscle, eIF4EBP3L might constitute a fast switch for recovery of atrophic muscle.

Other eIF4EBPs also appear to function in the reaction of muscle to distinct stresses. Stress induces Drosophila *eif4ebp* as a metabolic brake [86],[87]. Likewise, either *eIF4EBP1* ^−/−^ or *eIF4EBP1* ^−/−^ *;eIF4EBP2* ^−/−^ mice show defects in fat and muscle metabolism under stress conditions, but not under normal conditions [86],[88],[89]. Our data suggest that eIF4EBP3L acts as a metabolic brake preventing anabolism in inactive muscle, and also regulates cell growth by specific translational control in response to TORC1. The presence of high levels eIF4EBP3L sensitize muscle to TORC1, in agreement with the recent finding that eIF4E/eIF4EBP ratio is a key determinant of TORC1 action [90]. Activity, nutritional status, and other factors influencing TORC1 activity would be expected to have stronger effects when eIF4EBPs are up-regulated. Strikingly, many manipulations that trigger either muscle growth or atrophy alter expression of an eIF4EBP [78],[80],[91],[92]. In the brain, eIF4EBP2 appears to act downstream of TORC1 signaling to control the translation of specific mRNAs involved in synaptogenesis and linked to autism [5]. As there are at least three eIF4EBP genes in all vertebrates examined, it is possible that each becomes a gatekeeper of TORC1 in response to distinct stresses, and could then select which particular mRNAs become TORC1 targets.

### Mef2ca as a Translationally Controlled Target

The specific regulation of *mef2ca* mRNA translation begs the question of the role of Mef2 in growing muscle. Myofibril assembly is critically dependent upon Mef2 activity, which seems to be particularly important for thick filament biogenesis [41],[46]. We show that Mef2ca by itself is essential for normal fiber growth. *Mef2ca* mRNA first accumulates as muscle undergoes terminal differentiation [41], and we show that myosin is reduced and myofibrilogenesis is inefficient in *mef2ca* mutants. The additional presence of Mef2d, Mef2cb, and Mef2aa in skeletal muscle may contribute to overall Mef2 activity, permitting lower but significant rates of myofibrilogenesis in *mef2ca* mutants [44]. Up-regulation of Mef2 level by electrical activity will contribute positively to myofibrilogenesis. Loss of Mef2ca alone has less effect upon myofibrilogenesis than loss of activity. It is likely that other translational and/or transcriptional targets of activity, possibly including Mef2d, contribute to this difference.

The Mef2 protein is itself a transcription factor activated by muscle electrical activity via calcineurin and inhibited in cancer-induced muscle wasting [93],[94]. Our discovery of a TORC1-4EBP-Mef2 pathway that regulates muscle mass reveals an additional level of activity-dependent regulation of Mef2. The TORC1-4EBP-Mef2 pathway might be involved in other muscle-wasting conditions that affect TOR activity, such as fasting, ageing, and cachexia. By showing that Mef2c regulates muscle fiber growth regardless of any earlier role in myoblast terminal differentiation, our data support the view of Mef2 as a muscle homeostatic regulator [95],[96]. It will be interesting to determine whether translational regulation of human *MEF2C* is also important in the nervous system, where MEF2C is associated with autism and synaptic regulation [97].

In the heart, Mef2c is required for cardiomyocyte differentiation and expression persists in differentiated cardiomyocytes [44],[98]. Our finding of activity-dependent accumulation of Mef2 in skeletal muscle suggests a mechanism that could regulate heart muscle cell size and/or function. At later developmental stages, Mef2a becomes a major Mef2 required for normal heart development and prevention of heart attack in mouse [99] and zebrafish [42],[44],[100]. The precise role of Mef2a and other Mef2s in the adult heart and skeletal muscle is unclear, but our data suggest a role in controlling the balance between anabolism and catabolism.

Strikingly, both murine *Mef2c* and zebrafish *mef2ca* mRNAs accumulate at muscle fiber ends [41],[101]. We speculate that this mRNA localization may facilitate their translational regulation by activity-dependent signals, including, but not necessarily restricted to, TORC1-4EBP. Such regulation would provide a novel paradigm in regulation of muscle gene expression, whereby changing translation sends a signal back to the nucleus to regulate transcription. In this scenario, the gatekeeper function of eIF4EBP3L could control the sensitivity of localized mRNAs to local signals generated at the muscle fiber end. For example, mechanical force, transmitted from muscle to its attachments at the fiber end, could trigger signals mediating the effects of activity on translation. Such regulation via TORC1 may contribute to the potent hypertrophic effect of high-force stimuli on muscle [102],[103]. Regulated translation of transcription factors could mediate mechanical or other signals elsewhere in biology.

There is great societal importance to determining the mechanisms by which physical activity enhances well-being both in the elderly and throughout life. Our findings show that one effect of activity is to control translation of specific muscle proteins that themselves influence muscle growth. As a major metabolic tissue constituting almost half our body mass, skeletal muscle and its energy balance are increasingly understood to be a significant endocrine regulator of whole body physiology. It will be important to determine whether and at what life stages the pathway we have revealed regulates the response of human muscle to activity.

## Materials and Methods

### Ethics Statement

All work described was performed under licenses conforming to UK Animals (Scientific Procedures) Act 1986.

### Fish Husbandry and Embryo Manipulation

D. rerio wild-type and *mef2ca^tn213^* [104], *chrnd^sb13^* [39] mutants were maintained on Tübingen background and reared according to [105]. 0.016% MS222 (3-amino benzoic acid ethyl ester, 0.64 mM) was added to embryo medium for 17–24 h. Embryos were injected at 1–2 cell stage with 1–2 ng of MOs against *eif4ebp3l*; exon1-intron1 junction (BP3L MO1) and start codon (BP3L MO2) or with standard control MO (Gene Tools) or with 100 pg *eif4ebp3l* RNA made using Ambion Megascript kit from CloneJET plasmid containing full-length zebrafish *eif4ebp3l*. The plasmids pCS2-ZF-HSP70-5A3L-IRES-GFP or empty vector were injected at 1 cell stage. Heat shock of 39°C for 2 h was applied at 30 hpf and 48 hpf, and myofiber width was measured at 54 hpf. Cell Trace BODIPY (#C34556 Invitrogen) was added during the second heat shock, washed, and imaged live 4 h later. Dechorionated 2 dpf embryos were treated for 6–8 h with 1–5 µM rapamycin or 100 nM MG132 (Cayman #13697).

### Cloning and mutagenesis

*eIf4ebp3l* cDNA was PCR cloned into CloneJET pJET1.2 (Thermo Scientific) using primers listed in Table S1. The 5A3L mutant was generated by substituting T33,T42,T46,S61,T66 to alanine using QuikChange II Site-Directed Mutagenesis Kit #200523 (Agilent Technologies) with primers listed in Table S1. The 5A3L mutant *eIf4ebp3l* was cloned into XbaI/SalI digested *hsp70-4-MCS-IRES-mGFP6*, kindly donated by S. Gerety and D. Wilkinson [41].

### Immunodetection

Immunodetection in whole embryos was as previously described [41]. Confocal stacks were collected on a Zeiss LSMExciter confocal microscope and signal intensity was calculated using Volocity software. All embryos are shown in lateral view, anterior to left, dorsal to top. Myofibrillar bundle width was measured on slow MyHC in 10 fibers in somite 17 by assessing transverse dorsoventral width of three well-bundled regions in anterior, middle, and posterior of each fiber from maximal intensity projections. To ensure fair fiber size comparison in mosaic analyses, the myofibrils of GFP^+^ fibers were compared with the four immediately surrounding GFP^−^ slow fibers. For Westerns, protein extracts were made from 20–50 embryos using SDS loading buffer. Samples were incubated for 5 min at 100°C, sonicated, and stored at −20°C. Proteins from ∼5 embryo-equivalents were separated by PAGE, blotted to PVDF membrane which was blocked with 5% low fat milk for 1 h RT, incubated overnight with primary antibody and for 1 h RT with secondary antibody, developed with ECL Super Signal® #34080 (Thermo Scientific), and assessed on ImageJ software. Primary antibodies used were: pS6 #5364, S6 #2317, p4EBP^T37^ #2855, 4EBP^T46^ #4923 (Cell Signalling), Vinculin #V9131, Actin #A2066 (Sigma), Mef2c #55913 (Anaspec), Mef2 C-21 #SC313, MyoD C-20 #SC302 (Santa Cruz), SQSTM1 #ab56416, Tropomyosin #ab7786 (Abcam), general MyHC (A4.1025) [106], slow MyHC (F59) [31], MyLC #F310 (DSHB), and Myosin binding protein C (MyBPC, a kind gift of E. Ehler).

### RNA detection

In situ RNA hybridization was performed as previously described [41]. Probes for *eif4ebp1*, *eif4ebp2*, *eif4ebp3*, and *ei4ebp3l* were prepared by T7/T3 RNA polymerase with primers listed in Table S1. Other probes were as described: *mef2ca*, *mef2d* [42], *mef2aa, mef2cb* [44], *mef2ab* (NCBI AL918279), and *mef2b* (Exelixis 3573227, NCBI JX292158). qPCR analysis used SYBR green MESA Blue (RT-SY2X-03 WOUB, Eurogentec) with specific primers listed in Table S1 and were performed in triplicate to ensure reproducibility. cDNA was generated from 20 embryos using Invitrogen SuperScript III.

### Polysomal fractionation

We adjusted the protocol of [107] to zebrafish. Briefly, dechorionated embryos were incubated with 400 µg/ml cycloheximide for 10 min, snap frozen in liquid nitrogen, pulverized by pestle and mortar, and stored at −80°C. Powder was resuspended in 500 µl lysis buffer/70 embryos and the sample pipetted 10 times. Nuclei were removed by centrifugation (12,000× *g*, 10 s, 4°C). Supernatant was supplemented with 250 µl of extraction buffer and centrifuged (12,000× *g*, 5 min, 4°C) to remove mitochondria and membranous debris. 75 µl (1/10th) of the supernatant was stored at −80°C for total RNA. Remaining supernatant was layered onto a 10 ml linear 10%–50% sucrose gradient and ultracentrifuged for 2 h in Beckman SW40Ti at 38,000 rpm at 4°C, brake off. Samples were collected passing a UV 254 nm detector (Gilson UV/VIS-155) to detect the polysome profile and fractionated, generally into three ∼4 ml fractions. Samples were incubated with 200 µl/ml proteinase K, 1% SDS, 10 mM EDTA pH 8, for 30 min at 37°C. RNA was recovered by 1∶1 phenol∶chloroform extraction followed by ethanol precipitation with glycogen. RNA pellet was washed overnight with 2 M LiCl, and resuspended in 500 µl RNAse free water, treated with DNAse I for 30 min, ethanol precipitated, and 200 ng RNA was used per reverse transcription reaction.

## Supporting Information

Figure S1 ***chrnd^sb13/sb13^*** **mutant lacks acetylcholine receptors.** Zebrafish *chrnd^sb13^* mutant 48 hpf embryos were stained with α-bungarotoxin-Alexa Fluor 555 (Invitrogen), which binds to the acetylcholine receptor. Note the lack of signal in mutant at both neuromuscular junction (arrows) and myotendinous junction (arrowheads). Bar = 40 µm.
(TIF)

Figure S2 **Three Mef2 mRNAs accumulate in 48 hpf muscle.** In situ mRNA hybridization for *mef2* family members in 48 hpf zebrafish trunk and tail.
(TIF)

Figure S3 **Controls for proteasome and autophagy analysis.** (A) Western analysis for p53 on zebrafish treated with MG132 or vehicle control from 30 hpf to 48 hpf. (B) QPCR of 48 hpf embryos treated for 17 h with MS222 or inactive due to *chnrd* mutation revealed no increase in *sqstm1* mRNA. (C) Western analysis showing that Mef2c reduction induced by MS222 from 31 hpf to 48 hpf occurs in the presence of the lysosomal inhibitor chloroquine (50 µm).
(TIF)

Figure S4 **Polysome profiling of whole zebrafish embryos.** Embryos at 48 hpf were subjected to polysome profiling in the presence or absence of EDTA (from the lysis step onwards) or following 6 h cycloheximide treatment (400 µg/ml).
(TIF)

Figure S5 **Inactivity does not alter ribosomal RNA content.** (A) Bioanalyzer scans of RNA samples prepared from equal numbers of control or MS222-treated embryos. (B) Quantification of ribosomal RNA recovered per embryo.
(TIF)

Figure S6 **Inactivity reduces myofibrilogenesis more significantly than rapamycin.** Zebrafish embryos were treated with MS222, rapamycin, both, or vehicle control from 31–32 hpf and analyzed at 48 hpf. (A) Immunofluorescence of slow MyHC on whole-mount embryos treated with either MS222 or rapamycin for 17 h. Note that brightness was enhanced on MS222 image to show myofibril bundles. (B) Effect of rapamycin on pS6 immunoreactivity in muscle. (C) Quantification of slow fiber myofibril bundle width. Mean ± SEM (*n* = 8 embryos in each condition, 10 fibers measured per embryo). (D) Western analysis of Tropomyosin, Actin, and Fast MyLC in the presence of MS222, rapamycin, or both.
(TIF)

Figure S7 **Rapamycin inhibits TOR in embryonic zebrafish.** Western analysis of 48 hpf embryos treated with rapamycin for 6 h, showing the reduction in the phosphorylated forms of ribosomal protein S6 and eIF4EBPs. The ratio of phospho4EBP∶total 4EBP normalized to control is shown below each lane.
(TIF)

Figure S8 **Zebrafish eIF4EBP3L is a conserved eIF4EBP induced in inactive skeletal muscle.** (A–C) Comparison of human and zebrafish eIF4EBPs using MegAlign Jotun-Hein method (DNAstar v10, Lasergene) to align eIF4EBP sequences from ENSEMBL Zv9 and Hu GRCh37. (A) Tree view from MegAlign. (B) Amino acid identity and diversity between human and zebrafish eIF4EBPs. (C) Alignment of human and zebrafish eIF4EBPs. TOR phosphorylation sites are marked with red arrows, eIF4E binding site with black line, and residues mutated in 5A3L are marked red. (D) Expression of zebrafish eIF4EBPs at 48 hpf by in situ mRNA hybridization. Top row shows expression in head; middle row in somites; bottom row in somites of sibling embryos treated for 24 h with MS222 revealing a tissue-specific increase in *eif4ebp3l* mRNA. Arrow indicates previously described *eif4ebp3* pancreas expression. Bars = 200 µm.
(TIF)

Figure S9 **RNA analysis of** ***chrnd^sb13/sb13^*** **mutant and siblings.** cDNA from 48 hpf zebrafish *chrnd* ***^sb13/sb13^*** mutants and siblings, sorted by fully penetrant immotility phenotype, was assayed by qPCR for E3 ligase atrogenes (A) and for *mef2ca* and *smyhc1* (B).
(TIF)

Figure S10 **Overexpression of eIF4EBP3L in zebrafish.** (A) Levels of overexpression of *eif4ebp3l* are within the physiological range. In situ RNA hybridization (upper panel) and qPCR quantification (lower panel) at 48 hpf of embryos injected with *eif4ebp3l* RNA. (B) No obvious effect of overexpression of constitutively active eIF4EBP3L (5A3L) on nonmuscle cells. Zebrafish embryos were injected with plasmid encoding a heat-shock-driven 5A3L-IRES-GFP. Following heat shock and BODIPY incubation, embryos were scanned live. Control GFP-negative cells are outlined in epidermis to show similar size.
(TIF)

Figure S11 **Knockdown of eIF4EBP3L prevents muscle-activity-dependent** ***mef2ca*** **translation regulation.** Embryos injected with control MO or splice-blocking MO against *eif4ebp3l* (eIF4EBP3L MO) were grown from 32–48 hpf in the presence or absence of MS222. All embryos were co-injected with p53 MO to prevent cell death caused by suspected off-target effects of the eIF4EBP3L MO. Embryo morphology was normal in all groups. Polysomal fractionation, RNA isolation, and cDNA synthesis was followed by triplicate qPCR for *mef2ca* or *dmd* mRNAs on total RNA and polysomal fractions. Results were corrected for RNA yield and normalized to the control MO and are presented as mean ± sem. Note that the larger sem with eIF4EBP3L MO reflects high variability within triplicates, with no consistent effect of MS222 between two biological replicates.
(TIF)

Table S1 **Primers and Morpholino antisense olignucleotides.**
(DOCX)

Table S2 **mRNA folds change following inactivity (MS222) in subpolysomal fraction.**
(DOCX)

Table S3 **Pyrimidine-Rich Translational Element (PRTE) within 5′ UTRs.**
(DOCX)

## Abbreviations

dpf: days postfertilization
hpf: hours postfertilization
MS222: 3-amino benzoic acid ethyl ester
MO: morpholino antisense oligonucleotide
TOR: target of rapamycin
TORC1: target of rapamycin complex 1
