# Supplementary figures and images for: eIF4EBP3L Acts as a Gatekeeper of TORC1 In Activity-Dependent Muscle Growth by Specifically Regulating Mef2ca Translational Initiation
Source: PLoS Biol. 2013 Oct 15;11(10):e1001679. doi: 10.1371/journal.pbio.1001679 (PMC3797031; doi:10.1371/journal.pbio.1001679)

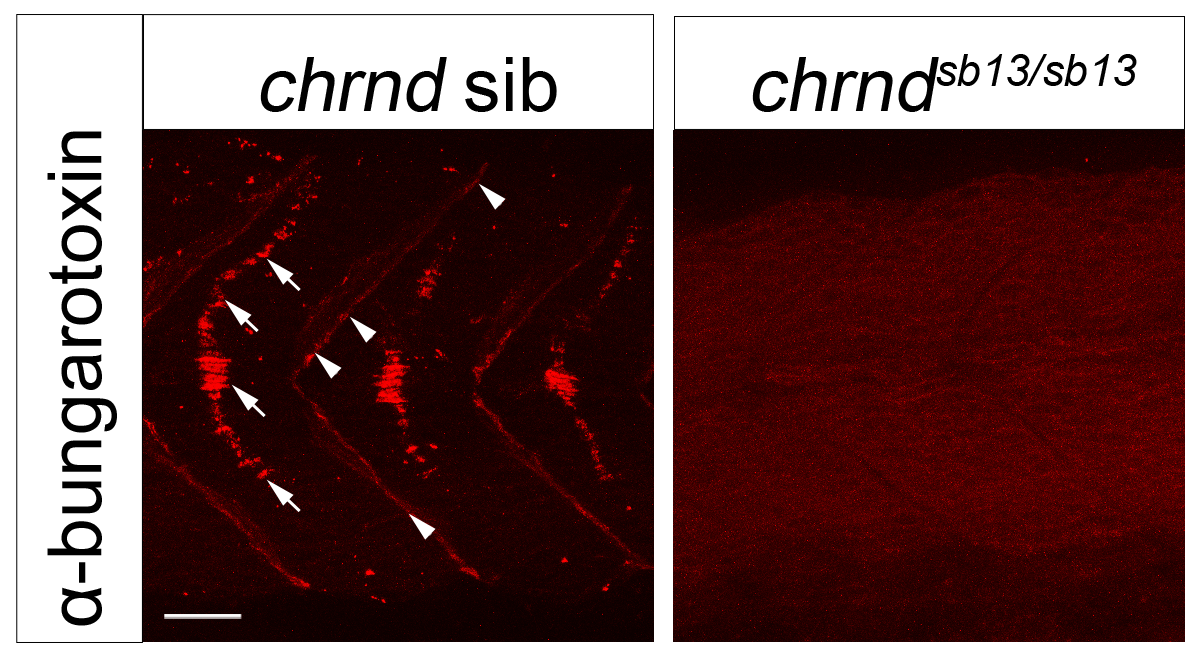

Supplement: Figure S1 — chrndsb13/sb13 mutant lacks acetylcholine receptors. Zebrafish chrndsb13 mutant 48 hpf embryos were stained with α-bungarotoxin-Alexa Fluor 555 (Invitrogen), which binds to the acetylcholine receptor. Note the lack of signal in mutant at both neuromuscular junction (arrows) and myotendinous junction (arrowheads). Bar = 40 µm. (TIF) [file pbio.1001679.s001.tif]

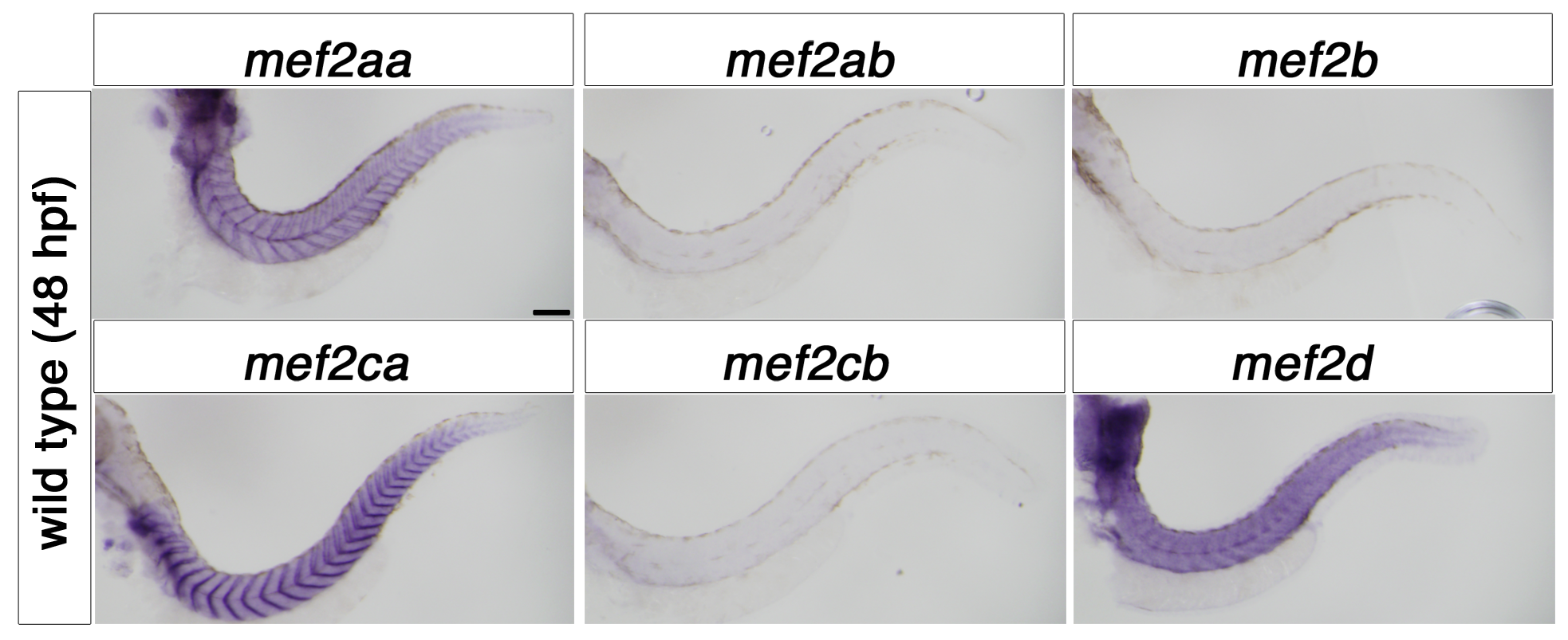

Supplement: Figure S2 — Three Mef2 mRNAs accumulate in 48 hpf muscle. In situ mRNA hybridization for mef2 family members in 48 hpf zebrafish trunk and tail. (TIF) [file pbio.1001679.s002.tif]

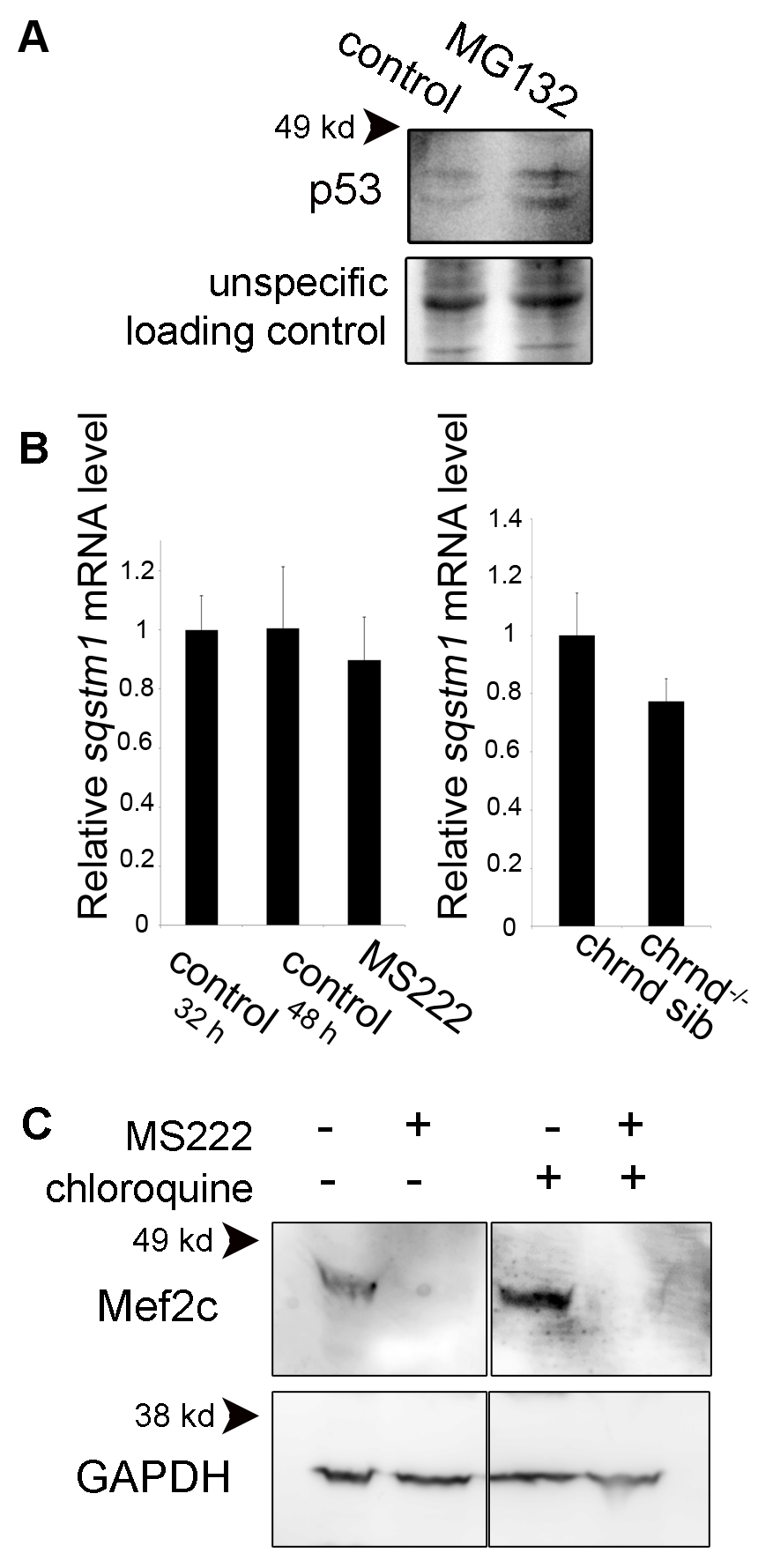

Supplement: Figure S3 — Controls for proteasome and autophagy analysis. (A) Western analysis for p53 on zebrafish treated with MG132 or vehicle control from 30 hpf to 48 hpf. (B) QPCR of 48 hpf embryos treated for 17 h with MS222 or inactive due to chnrd mutation revealed no increase in sqstm1 mRNA. (C) Western analysis showing that Mef2c reduction induced by MS222 from 31 hpf to 48 hpf occurs in the presence of the lysosomal inhibitor chloroquine (50 µm). (TIF) [file pbio.1001679.s003.tif]

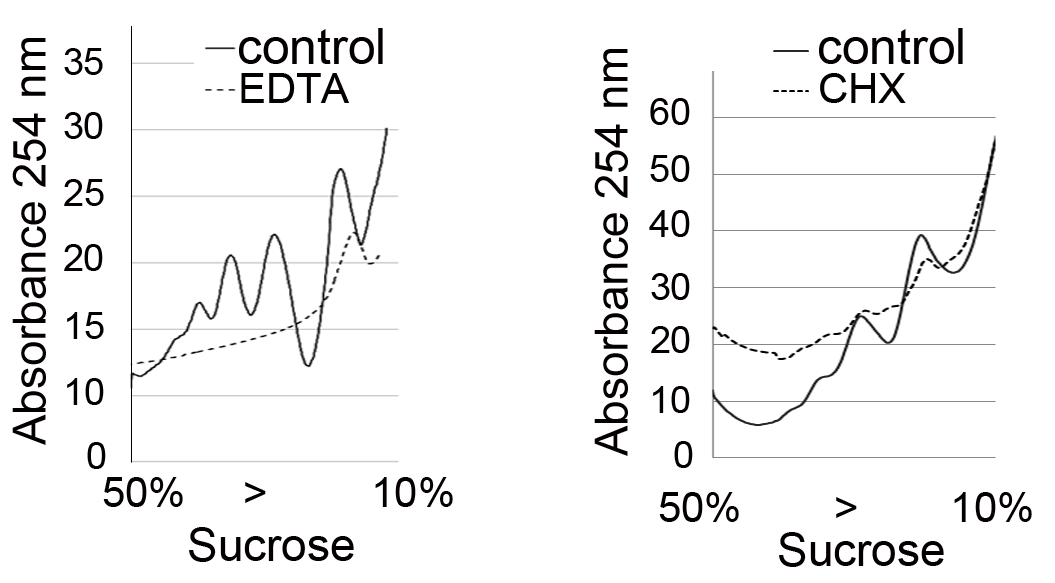

Supplement: Figure S4 — Polysome profiling of whole zebrafish embryos. Embryos at 48 hpf were subjected to polysome profiling in the presence or absence of EDTA (from the lysis step onwards) or following 6 h cycloheximide treatment (400 µg/ml). (TIF) [file pbio.1001679.s004.tif]

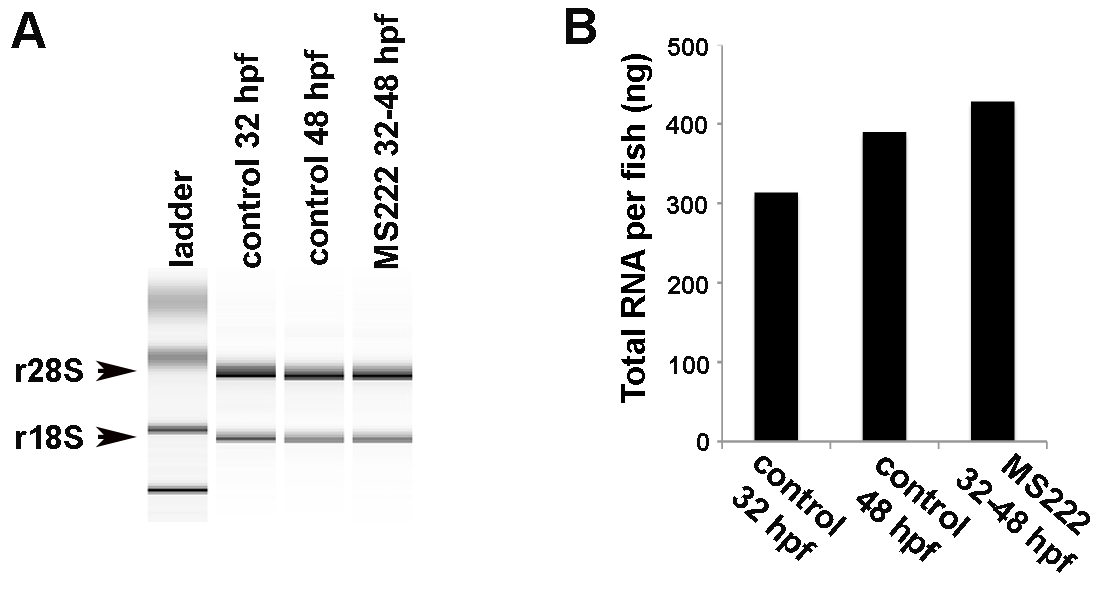

Supplement: Figure S5 — Inactivity does not alter ribosomal RNA content. (A) Bioanalyzer scans of RNA samples prepared from equal numbers of control or MS222-treated embryos. (B) Quantification of ribosomal RNA recovered per embryo. (TIF) [file pbio.1001679.s005.tif]

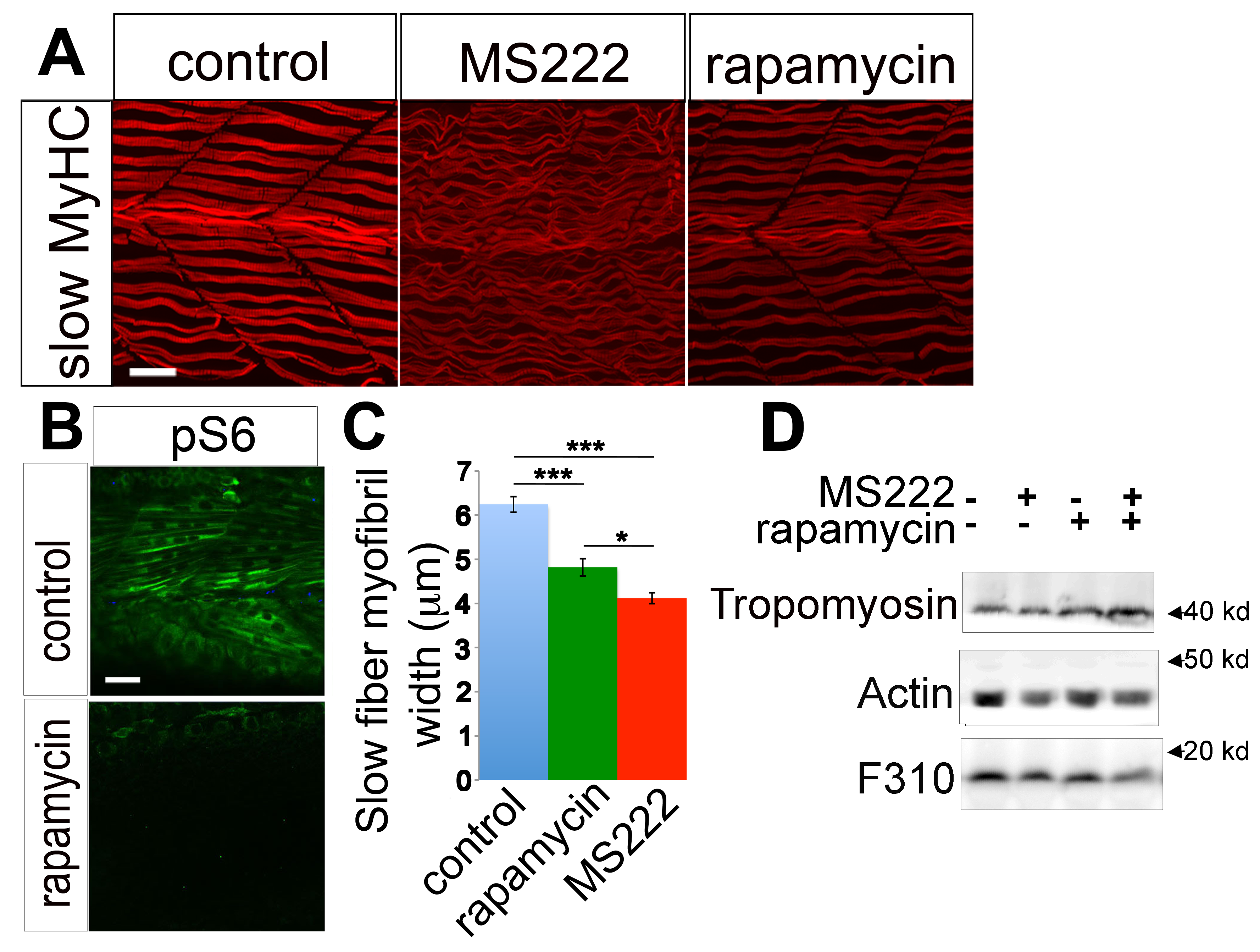

Supplement: Figure S6 — Inactivity reduces myofibrilogenesis more significantly than rapamycin. Zebrafish embryos were treated with MS222, rapamycin, both, or vehicle control from 31–32 hpf and analyzed at 48 hpf. (A) Immunofluorescence of slow MyHC on whole-mount embryos treated with either MS222 or rapamycin for 17 h. Note that brightness was enhanced on MS222 image to show myofibril bundles. (B) Effect of rapamycin on pS6 immunoreactivity in muscle. (C) Quantification of slow fiber myofibril bundle width. Mean ± SEM (n = 8 embryos in each condition, 10 fibers measured per embryo). (D) Western analysis of Tropomyosin, Actin, and Fast MyLC in the presence of MS222, rapamycin, or both. (TIF) [file pbio.1001679.s006.tif]

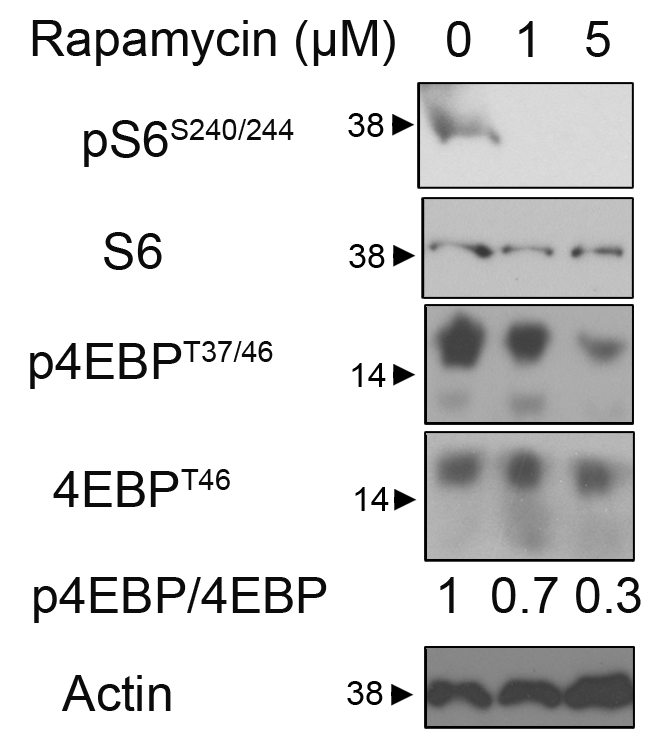

Supplement: Figure S7 — Rapamycin inhibits TOR in embryonic zebrafish. Western analysis of 48 hpf embryos treated with rapamycin for 6 h, showing the reduction in the phosphorylated forms of ribosomal protein S6 and eIF4EBPs. The ratio of phospho4EBP∶total 4EBP normalized to control is shown below each lane. (TIF) [file pbio.1001679.s007.tif]

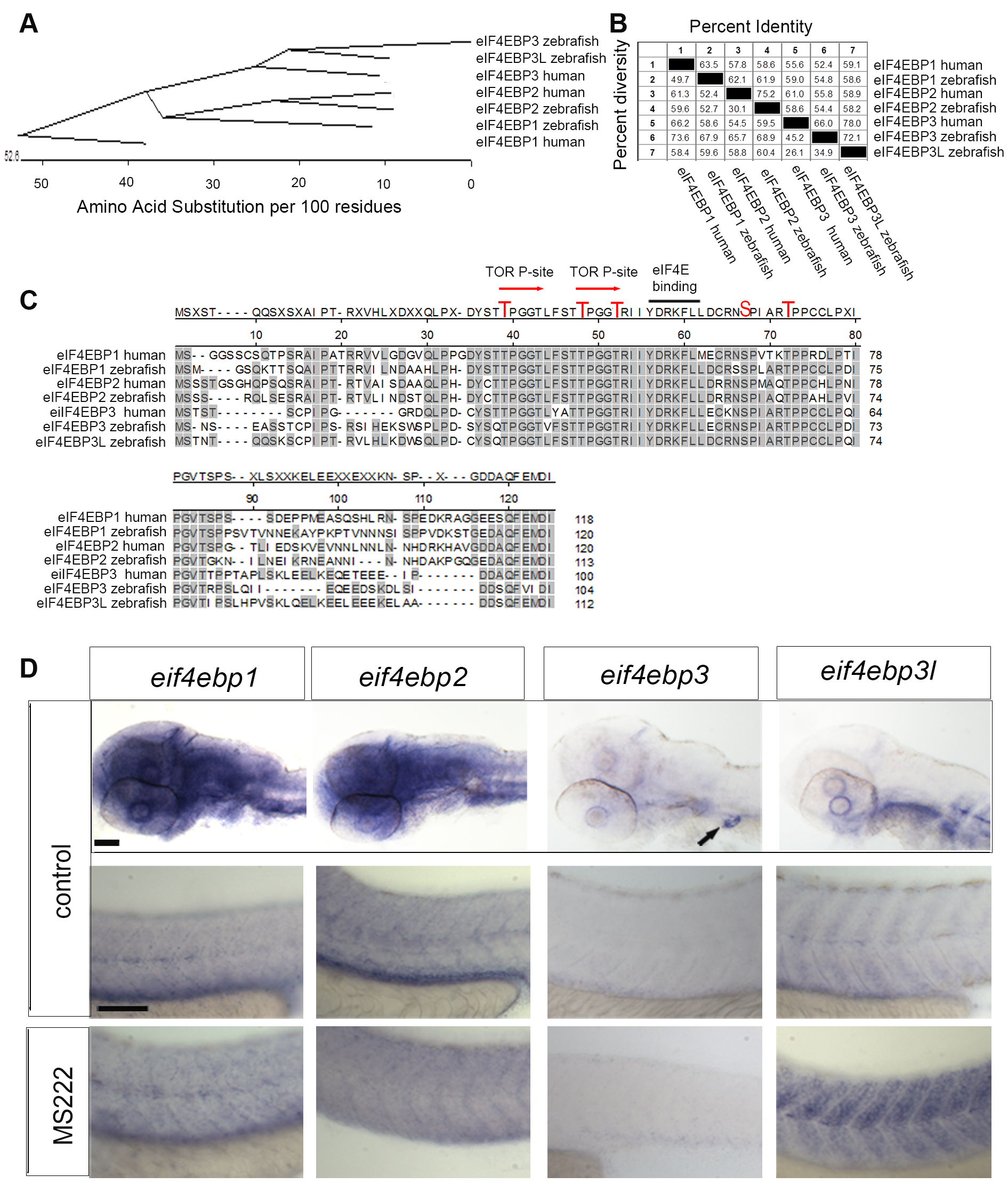

Supplement: Figure S8 — Zebrafish eIF4EBP3L is a conserved eIF4EBP induced in inactive skeletal muscle. (A–C) Comparison of human and zebrafish eIF4EBPs using MegAlign Jotun-Hein method (DNAstar v10, Lasergene) to align eIF4EBP sequences from ENSEMBL Zv9 and Hu GRCh37. (A) Tree view from MegAlign. (B) Amino acid identity and diversity between human and zebrafish eIF4EBPs. (C) Alignment of human and zebrafish eIF4EBPs. TOR phosphorylation sites are marked with red arrows, eIF4E binding site with black line, and residues mutated in 5A3L are marked red. (D) Expression of zebrafish eIF4EBPs at 48 hpf by in situ mRNA hybridization. Top row shows expression in head; middle row in somites; bottom row in somites of sibling embryos treated for 24 h with MS222 revealing a tissue-specific increase in eif4ebp3l mRNA. Arrow indicates previously described eif4ebp3 pancreas expression. Bars = 200 µm. (TIF) [file pbio.1001679.s008.tif]

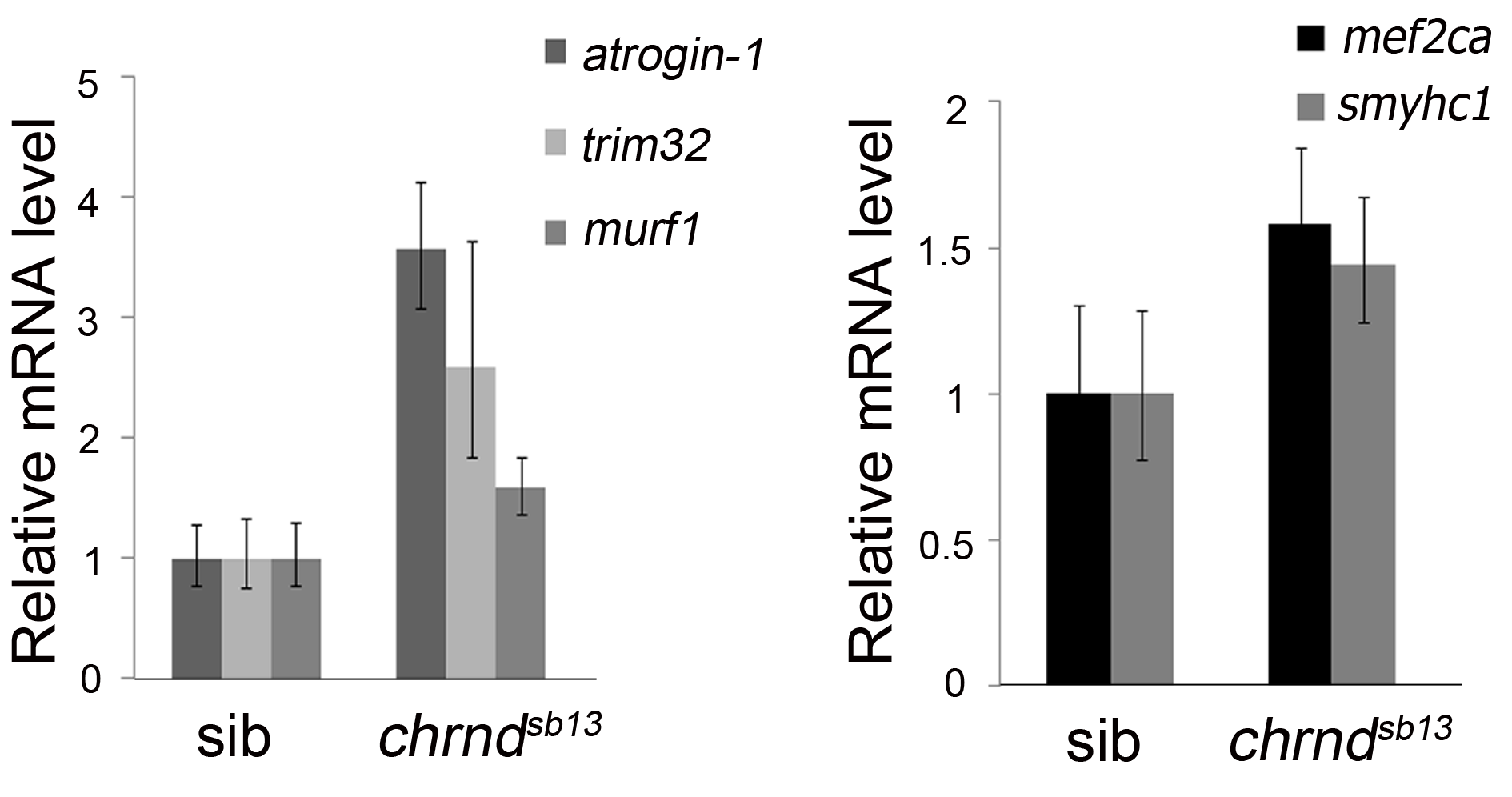

Supplement: Figure S9 — RNA analysis of chrndsb13/sb13 mutant and siblings. cDNA from 48 hpf zebrafish chrnd sb13/sb13 mutants and siblings, sorted by fully penetrant immotility phenotype, was assayed by qPCR for E3 ligase atrogenes (A) and for mef2ca and smyhc1 (B). (TIF) [file pbio.1001679.s009.tif]

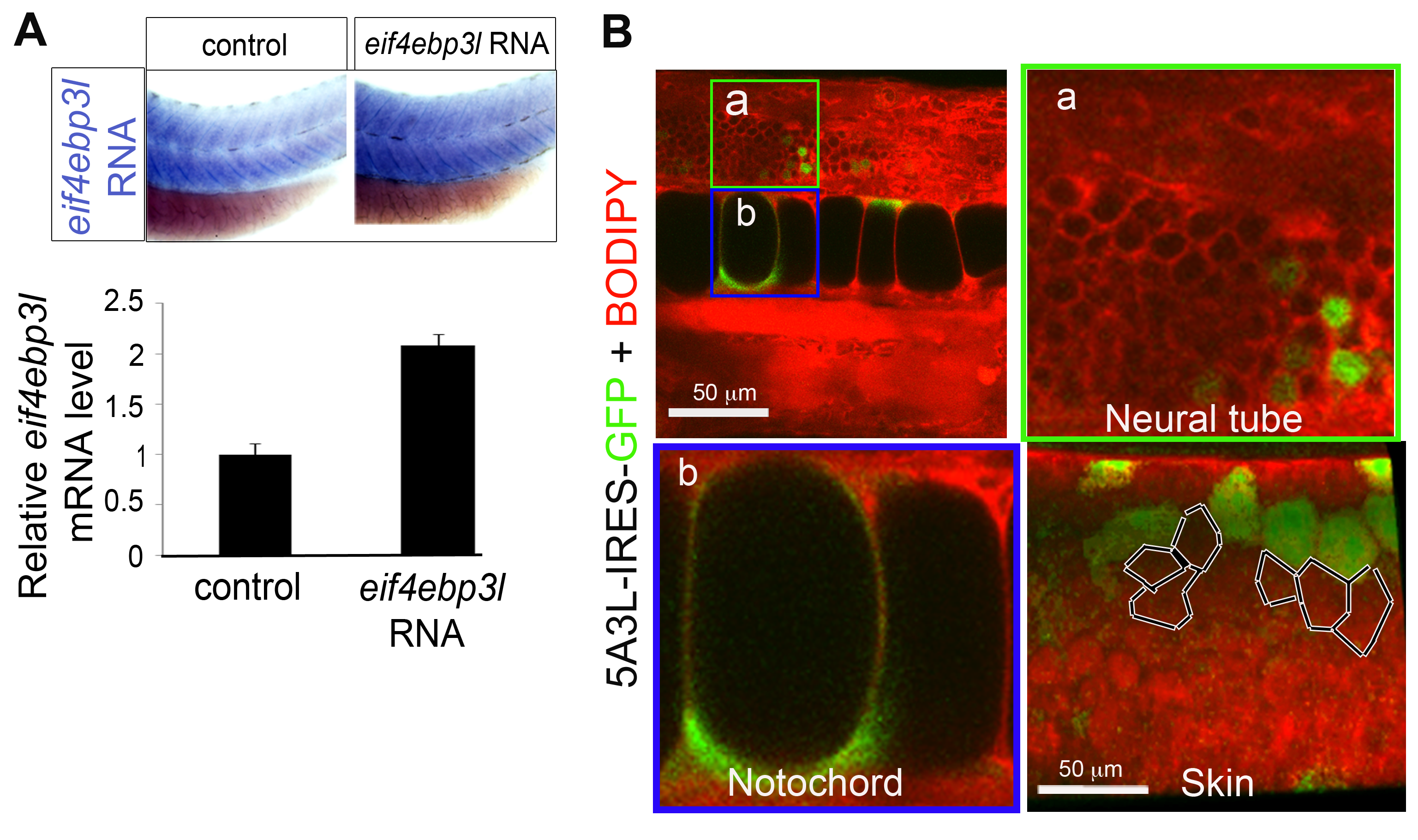

Supplement: Figure S10 — Overexpression of eIF4EBP3L in zebrafish. (A) Levels of overexpression of eif4ebp3l are within the physiological range. In situ RNA hybridization (upper panel) and qPCR quantification (lower panel) at 48 hpf of embryos injected with eif4ebp3l RNA. (B) No obvious effect of overexpression of constitutively active eIF4EBP3L (5A3L) on nonmuscle cells. Zebrafish embryos were injected with plasmid encoding a heat-shock-driven 5A3L-IRES-GFP. Following heat shock and BODIPY incubation, embryos were scanned live. Control GFP-negative cells are outlined in epidermis to show similar size. (TIF) [file pbio.1001679.s010.tif]

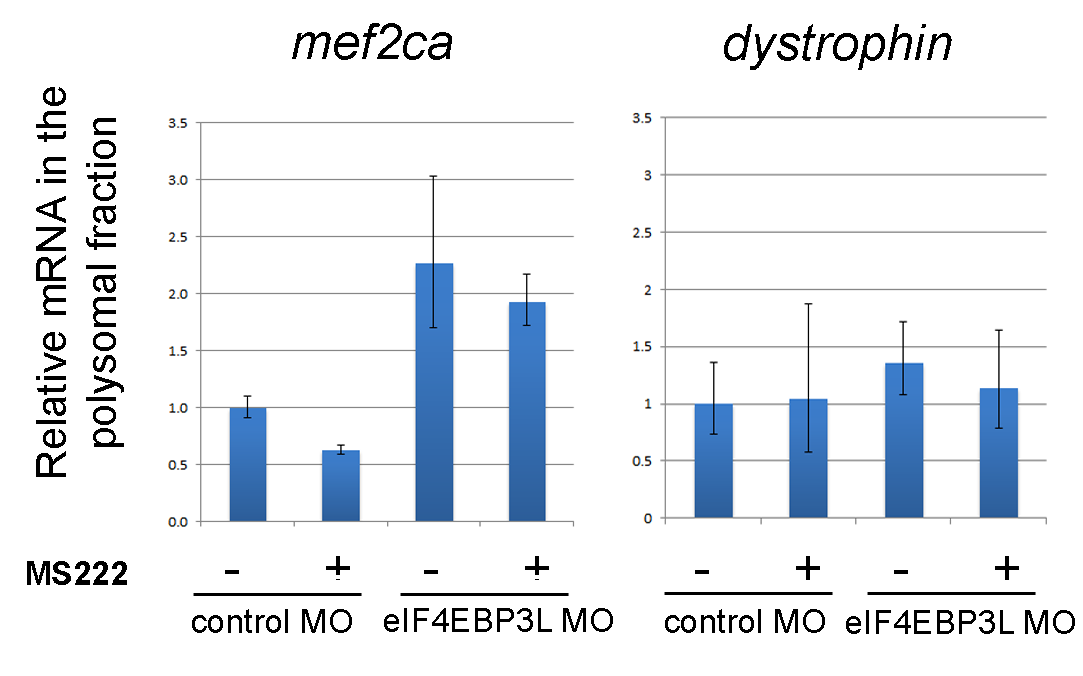

Supplement: Figure S11 — Knockdown of eIF4EBP3L prevents muscle-activity-dependent mef2ca translation regulation. Embryos injected with control MO or splice-blocking MO against eif4ebp3l (eIF4EBP3L MO) were grown from 32–48 hpf in the presence or absence of MS222. All embryos were co-injected with p53 MO to prevent cell death caused by suspected off-target effects of the eIF4EBP3L MO. Embryo morphology was normal in all groups. Polysomal fractionation, RNA isolation, and cDNA synthesis was followed by triplicate qPCR for mef2ca or dmd mRNAs on total RNA and polysomal fractions. Results were corrected for RNA yield and normalized to the control MO and are presented as mean ± sem. Note that the larger sem with eIF4EBP3L MO reflects high variability within triplicates, with no consistent effect of MS222 between two biological replicates. (TIF) [file pbio.1001679.s011.tif]
