# Supplementary material for: eIF4EBP3L Acts as a Gatekeeper of TORC1 In Activity-Dependent Muscle Growth by Specifically Regulating Mef2ca Translational Initiation
Source: PLoS Biol. 2013 Oct 15;11(10):e1001679. doi: 10.1371/journal.pbio.1001679 (PMC3797031; doi:10.1371/journal.pbio.1001679)
Supplement: Table S1 — Primers and Morpholino antisense olignucleotides. (DOCX) [file pbio.1001679.s012.docx]

**Supplementary Table S1. Primers and Morpholino antisense olignucleotides.**

| [ENSDARG00000041607](http://www.ensembl.org/Danio_rerio/Gene/Summary?g=ENSDARG00000041607) | | ***eif4ebp3l*** |
| --- | --- | --- |
| ***eif4ebp3l* cloning primers** | | Forward: ACATCACGAGAGCAGCACGCTAAA  Reverse: GCGGCCGCAGGCAAACGAAACACCAGGAAACC |
| ***eif4ebp3l* mutagenesis primers** | | T42,46A Forward: ACGCTCTTCTCCACCGCACCTGGCGGAGCC. |
|  |  | T42,46A Reverse: GTCGTAAATGATCCTGGCTCCGCCAGGTGC. |
|  |  | S61A+T66A Forward: TTCCTCCTGGACTGCAGGAACGCTCCGATCGCCCGGGCTCC |
|  |  | S61A+T66A Reverse: CTGGGGCAAACAGCAGGGTGGAGCCCGGGCGATCGGAGCGT |
|  |  | T33A Forward: GCTACAGTCAGGCCCCCGGGGGGAC  T33A Reverse: GTCCCCCCGGGGGCCTGACTGTAGC |
| **Probe primers** | | |
| [ENSDARG00000041607](http://www.ensembl.org/Danio_rerio/Gene/Summary?g=ENSDARG00000041607) | | ***eif4ebp3l***  Forward: ACATCACGAGAGCAGCACGCTAAA  Reverse: GGATCCATTAACCCTCACTAAAGGAGGCAAACGAAACACCAGGAAACC |
| [ENSDARG00000043608](http://www.ensembl.org/Danio_rerio/Gene/Summary?g=ENSDARG00000043608) | | ***eif4ebp1***  Forward: CCACAAACGGACAAGGTGCAAAGA  Reverse: GGATCCATTAACCCTCACTAAAGGATCAAACAAAGTGTGGCATGGCGG |
| [ENSDARG00000031819](http://www.ensembl.org/Danio_rerio/Gene/Summary?g=ENSDARG00000031819) | | ***eif4ebp2***  Forward: TCAACAACCATGATGCCAAGCCAG  Reverse: GGATCCATTAACCCTCACTAAAGGTGTGCGCTTATGAGACAGACCGAT |
| [ENSDARG00000054916](http://www.ensembl.org/Danio_rerio/Gene/Summary?g=ENSDARG00000054916) | | ***eif4ebp3***  Forward: AAACAGTGAAGCTTCGTCGACCTG  Reverse: GGATCCATTAACCCTCACTAAAGGCAGCCAACTGCTGATTTCTGGCAT |
| **qPCR primers** | |  |
| [ENSDARG00000037870](http://www.ensembl.org/Danio_rerio/Gene/Summary?g=ENSDARG00000037870) | | ***actinb2***  Forward: TACAGCTTCACCACCACAGC  Reverse: AAGGAAGGCTGGAAGAGAGC |
| [ENSDARG00000001431](http://www.ensembl.org/Danio_rerio/Gene/Summary?g=ENSDARG00000001431) | | ***actn3b (α-actinin3b)***  Forward: TCCTCAGGCAGGCCTTGTTTATCA  Reverse: TCACAATGAGGCGATCTGAAGGGT |
| [ENSDARG00000008487](http://www.ensembl.org/Danio_rerio/Gene/Summary?g=ENSDARG00000008487) | | ***dmd (dystrophin)***  Forward: ATCACAACAAGCAGCTGGAATCGC  Reverse: ACTGGACAGCTCATCATCACCCAT |
| [ENSDARG00000043608](http://www.ensembl.org/Danio_rerio/Gene/Summary?g=ENSDARG00000043608) | | ***eif4ebp1***  Forward: TCAGAAGACCACCAGTCAGGCAAT  Reverase: AGTCCAGCAGGAACTTTCGGTCAT |
| [ENSDARG00000031819](http://www.ensembl.org/Danio_rerio/Gene/Summary?g=ENSDARG00000031819) | | ***eif4ebp2***  Forward: TAAGTTCCTGTTGGACCGGCGTAA  Reverse: TTCAGGATGTTCTTGCCCGTCACT |
| [ENSDARG00000054916](http://www.ensembl.org/Danio_rerio/Gene/Summary?g=ENSDARG00000054916) | | ***eif4ebp3***  Forward: AAACAGTGAAGCTTCGTCGACCTG  Reverse: AACACAGTTCCTCCTGGTGTCTGA |
| [ENSDARG00000041607](http://www.ensembl.org/Danio_rerio/Gene/Summary?g=ENSDARG00000041607) | | ***eif4ebp3l***  Forward: ACAAACACGCAGCAGAGCAAGA  Reverse: ATGTCCATCTCAAACTGGCTGTCG |
| [ENSDARG00000031756](http://www.ensembl.org/Danio_rerio/Gene/Summary?g=ENSDARG00000031756) | | ***mef2aa***  Forward: GCCCCCGCAAAACTTCTCC  Reverse: ACTTCGCAGGCATGACTTTACCAA |
| [ENSDARG00000029764](http://www.ensembl.org/Danio_rerio/Gene/Summary?g=ENSDARG00000029764) | | ***mef2ca***  Forward: TCCAGGGAACATGAGCAAGAACCT  Reverse: ACTGAGAGCTGCTGATTCGCTGAT |
| [ENSDARG00000009418](http://www.ensembl.org/Danio_rerio/Gene/Summary?g=ENSDARG00000009418) | | ***mef2cb***  Forward: ACTCGGACATAGTGGAGACCCTG  Reverse: TTCTTTGCAGGCCGTGGTGGG |
| [ENSDARG00000040237](http://www.ensembl.org/Danio_rerio/Gene/Summary?g=ENSDARG00000040237) | | ***mef2d***  Forward: AATCCAAGCAGTCTCGTCACCACT  Reverse: TTGTTGAGGTCACCACCCAGAAGA |
| [ENSDARG00000067990](http://www.ensembl.org/Danio_rerio/Gene/Summary?g=ENSDARG00000067990) | | ***myhz1.1***  Forward: CAACATTTCTGGCTGGCTGGACAA  Reverse: TCTGCATGGAACCACCCTTCTTCT |
| ENSDARG00000071430 | | ***smyhc1***  Forward: TGGAAAGTGCCCAGAAAGAGTCCA  Reverse: ATGCTCTTTCCGGTTTCTCCGAGT |
| [ENSDART00000140061](http://www.ensembl.org/Danio_rerio/Transcript/Summary?db=core;g=ENSDARG00000075014;r=14:14448389-14453802;t=ENSDART00000140061) | | ***sqstm1***  Forward: GGCTAACGTGGAGTACCTAAAG  Reverse: TTCCCTCATGCTCAACATCG |
| **Morpholinos eIF4EBP3L** | | |
|  | Splice BP3L-MO1: ATAGTGAGAGTGGGTCTTACCGCCA | |
|  | ATG BP3L-MO2: TTGTGGACATCGTGCGTCAAAATGC | |
