# Supplementary material for: eIF4EBP3L Acts as a Gatekeeper of TORC1 In Activity-Dependent Muscle Growth by Specifically Regulating Mef2ca Translational Initiation
Source: PLoS Biol. 2013 Oct 15;11(10):e1001679. doi: 10.1371/journal.pbio.1001679 (PMC3797031; doi:10.1371/journal.pbio.1001679)
Supplement: Table S2 — mRNA folds change following inactivity (MS222) in subpolysomal fraction. (DOCX) [file pbio.1001679.s013.docx]

**Table S2: mRNA folds change following inactivity (MS222) in subpolysomal fraction**

| **Gene** | **Sub polysomal** **inactive/active** |
| --- | --- |
| ***Dystrophin*** | 1.84 |
| ***actinin α3b*** | 1.11 |
| ***mef2ca*** | 2.078 |
| ***mef2d*** | 2.13 |
| ***smyhc1*** | 3.44 |
