# Supplementary material for: eIF4EBP3L Acts as a Gatekeeper of TORC1 In Activity-Dependent Muscle Growth by Specifically Regulating Mef2ca Translational Initiation
Source: PLoS Biol. 2013 Oct 15;11(10):e1001679. doi: 10.1371/journal.pbio.1001679 (PMC3797031; doi:10.1371/journal.pbio.1001679)
Supplement: Table S3 — Pyrimidine-Rich Translational Element (PRTE) within 5′ UTRs. (DOCX) [file pbio.1001679.s014.docx]

**Table S3: Pyrimidine-Rich Translational Element (PRTE) within 5′ UTRs.**

| mRNA | PRTE site within the 5’ UTR |
| --- | --- |
| ***dystrophin*** | - |
| ***α-actinin3b*** | - |
| ***eif4ebp3l*** | CTTTTTTTTCTT |
| ***mef2ca*** | CCTTCTCCCCTTC, TTTCTTTTCCTTT |
| ***mef2d*** | CTTTCTTTCTTCCTTCCCTCCTTCCTTTTTT |
| ***myhz1.1*** | - |
| ***smyhc1*** | - |

PRTE sites were found to be enriched in mTOR-4EBP1 gene targets [[1](#_ENREF_1)].
